# Hypoxia regulates overall mRNA homeostasis by inducing Met^1^-linked linear ubiquitination of AGO2 in cancer cells

**DOI:** 10.1038/s41467-021-25739-5

**Published:** 2021-09-13

**Authors:** Hailong Zhang, Xian Zhao, Yanmin Guo, Ran Chen, Jianfeng He, Lian Li, Zhe Qiang, Qianqian Yang, Xiaojia Liu, Caihu Huang, Runhui Lu, Jiayu Fang, Yingting Cao, Jiayi Huang, Yanli Wang, Jian Huang, Guo-Qiang Chen, Jinke Cheng, Jianxiu Yu

**Affiliations:** 1 State Key Laboratory of Oncogenes and Related Genes, Department of Biochemistry and Molecular Cell Biology & Shanghai Key Laboratory of Tumor Microenvironment and Inflammation, Shanghai Jiao Tong University School of Medicine, Shanghai, 200025 China; 2 Department of Pathophysiology, Key Laboratory of Cell Differentiation and Apoptosis of Chinese Ministry of Education, Shanghai Jiao Tong University School of Medicine, Shanghai, 200025 China

**Keywords:** RNA, Cancer, Ubiquitylation

## Abstract

Hypoxia is the most prominent feature in human solid tumors and induces activation of hypoxia-inducible factors and their downstream genes to promote cancer progression. However, whether and how hypoxia regulates overall mRNA homeostasis is unclear. Here we show that hypoxia inhibits global-mRNA decay in cancer cells. Mechanistically, hypoxia induces the interaction of AGO2 with LUBAC, the linear ubiquitin chain assembly complex, which co-localizes with miRNA-induced silencing complex and in turn catalyzes AGO2 occurring Met^1^-linked linear ubiquitination (M1-Ubi). A series of biochemical experiments reveal that M1-Ubi of AGO2 restrains miRNA-mediated gene silencing. Moreover, combination analyses of the AGO2-associated mRNA transcriptome by RIP-Seq and the mRNA transcriptome by RNA-Seq confirm that AGO2 M1-Ubi interferes miRNA-targeted mRNA recruiting to AGO2, and thereby facilitates accumulation of global mRNAs. By this mechanism, short-term hypoxia may protect overall mRNAs and enhances stress tolerance, whereas long-term hypoxia in tumor cells results in seriously changing the entire gene expression profile to drive cell malignant evolution.

## Abstract (web summary)

Met^1^-linked linear ubiquitination (M^1^-Ubi) is catalyzed by linear ubiquitin chain assembly complex (LUBAC). Here the authors show that Ago2 protein is M^1^-Ubi modified by LUBAC complex under hypoxia condition leading to less association of miRNA target mRNAs to Ago2 protein and de-repression of miRNA targets.

## Introduction

Post-transcriptional regulation of mRNA through mRNA deadenylation, translational repression and mRNA destabilization by miRNA-induced silencing complexes (miRISC) is an essential mechanism in modulating mRNA turnover^1^. AGO2, a key effector of miRISC, assembles with miRNAs to form the basic core of miRISC, and simultaneously recruits a flexible scaffold protein TNRC6/GW182 to make a bridge with the multiple downstream effectors, which prompt post-transcriptional silencing of complementary targeted mRNA^2^. In the case of more perfectly complementary base, miRNA-targeted mRNAs can be directly cleaved by AGO2 through its nuclease activity^3^. According to computational predictions and miRNA-targeted genome-wide identifications, more than half of protein-coding genes are quantitatively supervised by the miRNA regulation system^4^. Dysregulation of miRNA biogenesis and disruption of the miRNA pathway are found in human cancer samples, which provides the compelling evidence that miRNAs are crucially important for a wide variety of human diseases, including cancers^5,6^. Dissecting definitely mechanisms of miRNA biogenesis and miRNA-mediated gene silencing, especially in response to physiological or microenvironmental stress stimuli^7^, provides a promising therapeutic strategy for cancers.

Clinical and experimental evidence indicate that hypoxia is an important microenvironmental factor that promotes tumorigenesis and cancer development. Multiple molecular mechanisms underlying hypoxia promoting tumor evolution are previously described, including its inducing hypoxia-inducible factors (HIFs) and other transcription factors to activate gene transcriptions, enhancing specific oncogenic mRNA translation, as well as affecting the protein stability^8,9^. In addition, hypoxia also leads to inactivation of the demethylases KDM5A and KDM6A, which increase the levels of H3K4me3 and H3K27me3, respectively, and regulate the transcription of downstream genes^10^. However, whether hypoxia is involved in the regulation of the mRNA homeostasis has received little attention, although it is reported that hypoxia stabilizes some specific mRNAs by inhibiting the mechanism of nonsense-mediated RNA decay (NMD)^11^. In cancer cells, whether under hypoxia overall mRNA homeostasis is regulated in a NMD independent manner is not explored. In response to hypoxia, during tumorigenesis miRNA biogenesis is modulated through post-translational modifications of core processing enzymes of the miRNA pathway^12–14^, but whether hypoxia possesses the capacity in modulating miRNA functional efficiency and its underlying molecular mechanism is still unexplored.

Protein ubiquitination system has been emerged as a protein quantity detective and a huge cellular signaling transduction net for controlling signaling pathways^15^. The linear ubiquitin chain assembly complex (LUBAC), consisting of HOIP (RNF31), HOIL-1L (heme-oxidized IRP2 ubiquitin ligase 1 L) and SHARPIN (Shank-associated RH domain-interacting protein), has been identified as the unique ubiquitin ligase that specifically catalyzes Met^1^-linked linear polyubiquitin chain to its substrates, in which the N-terminal methionine (Met) of an ubiquitin can form a peptide bond with the C-terminal glycine (Gly) of another ubiquitin^16–19^. On the contrary, the Met^1^-linked linear polyubiquitination (termed as M1-Ubi) is negatively regulated by OTULIN (Gumby/FAM105B), CYLD and A20 with different mechanisms^20–23^. M1-Ubi endows with an essential role in activating inner inflammation and immune signaling^17–19^. HOIP or HOIL-1L deficiency in mice results in embryonic lethality at mid-gestation^24,25^ and SHARPIN deficiency in mice results in immunodeficiency and severe multiple-organs inflammation disease^26^, respectively. Native deficiency of either HOIP or HOIL-1L in human causes autoimmunity and inflammatory disease^27,28^. Meanwhile, native mutations of HOIP in human is closely related to lymphoma^29^. Since hundreds of linear polyubiquitin-modified substrates are identified by through mass spectrometry analysis combined with lysine-less internally tagged ubiquitin strategy^30^, M1-Ubi may be involved in variously cellular processes. Thus far, cellular signaling regulated by M1-Ubi in non-inflammation and non-immune signaling are still mostly unknown.

Here we find that hypoxia suppresses miRNA-mediated gene silencing, and the interactions of miRNA-targeted mRNAs with AGO2 are extensively inhibited under hypoxia stress. Intriguingly, we demonstrate that hypoxia induces the recruitment of LUBAC components HOIP and HOIL-1L to AGO2, thereby catalyzing M1-Ubi of AGO2. The M1-Ubi of AGO2 blocks its association with targeted mRNAs and subsequently decreases miRNA-mediated gene silencing, thus resulting in the accumulation of miRNA-targeted mRNAs. Thus, our studies reveal that M1-Ubi of AGO2 is an essential mechanism modulating mRNA turnover in the miRNA pathway in hypoxic cancer cells.

## Results

### Hypoxia impairs miRNA-targeted mRNA loading to AGO2

**Fig. 1:**
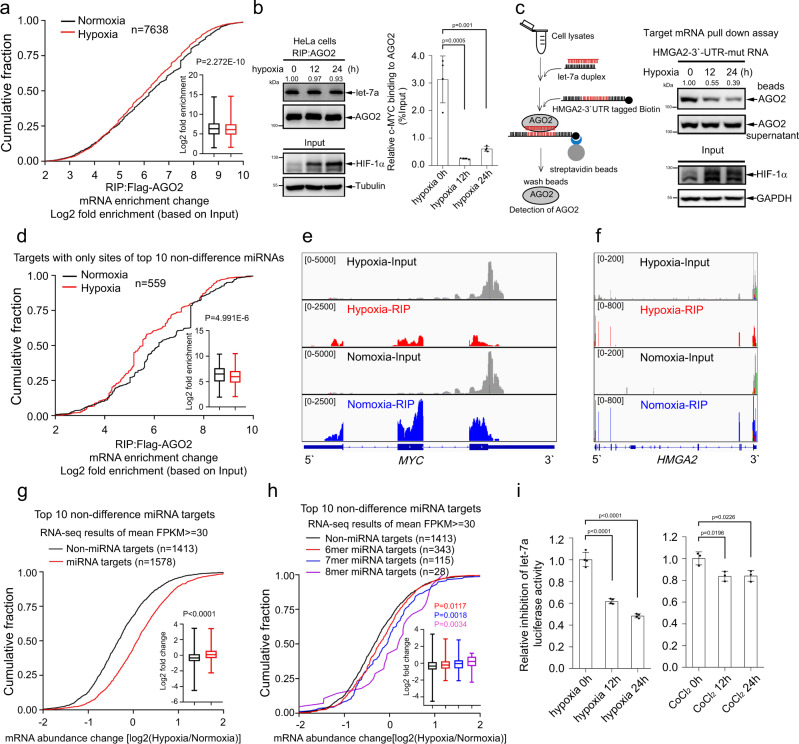
Hypoxia impairs miRNA-targeted mRNA loading to AGO2 and inhibits mRNA decay. **a** Hypoxia inhibited mRNA loading to AGO2. Cumulative fraction analysis of RIP-Seq for mRNA transcripts bound to AGO2 (*n* = 7638 mRNA transcripts) in stable HeLa cells expressing Flag-AGO2 under hypoxia (1% O_2_) for 24 h. **b**, **c** Hypoxia suppressed the interaction of let-7a-targeted *c-MYC* and *HMGA2*-3′-UTR-mut with AGO2. **b** HeLa cells treated with hypoxia were performed by RIP assay, let-7a and *c-MYC* associated with AGO2 were detected by northern blotting and qRT-PCR, respectively. Data were mean ± s.d., *n* = 4 biologically independent samples, and *P*-values were determined by unpaired two-sided t-test. **c** Cell lysates from 293 T cells treated with hypoxia were co-incubated with let-7a duplex mimics and biotin-tagged *HMGA2*-3′-UTR-mut for in vitro targeted mRNA pull-down assay. AGO2 associated with *HMGA2*-3′-UTR-mut was pulled down by streptavidin beads, and then detected by WB. **d**–**f** Hypoxia decreased the interaction of AGO2 with mRNAs targeted by only top 10 non-difference miRNAs. Cumulative fraction analysis of AGO2 bound mRNA transcripts (*n* = 559 mRNA transcripts) targeted by only top 10 non-difference miRNAs but not by hypoxia-reduced miRNAs in stable HeLa cells expressing Flag-AGO2 under hypoxia (**d**). Coverage plots showing the abundance of *MYC* (**e**) and *HMGA2* (**f**) mRNAs binding to AGO2 by RIP-Seq. **g**, **h** Hypoxia increased the accumulation of mRNA transcripts targeted by top 10 non-difference miRNAs (**g**) and their corresponding 6mer, 7mer and 8mer seed sequences with a single site (**h**). Cumulative fraction analyses were performed with RNA-Seq data in HeLa-Flag-AGO2 stable cell lines under normoxia and hypoxia. **i** Hypoxia inhibited the let-7a-miRISC activity. 293T cells were transfected psiCHECK2-4xlet-7a-BS and then treated with hypoxia or CoCl_2_ (300 μM) for 12 and 24 h. The let-7a-miRISC activity was measured by the dual-luciferase assay. Data were mean ± s.e.m., *n* = 4 (left panel) or *n* = 3 (right panel) biologically independent experiments, and *P*-values were determined by unpaired two-sided t-test. In box plots, the lines represent the median, first and third quartiles, the whiskers denote the minima and maxima; *P*-values were calculated using a two-sided Mann–Whitney U test for cumulative fraction analysis (**a**, **d**, **g**, **h**).

To investigate the role of hypoxia in modulating mRNAs loading to AGO2, RNA immunoprecipitation sequencing (RIP-Seq) of AGO2 were performed by using stable HeLa cells expressing Flag-AGO2, which were separately treated with normoxia (21% O_2_) and hypoxia (1% O_2_) for 24 h. 11,084 and 13,176 of mRNA transcripts associated with AGO2 were identified under normoxia and hypoxia conditions, respectively, and there were 7638 mRNA transcripts overlapping between normoxia (68.9%) and hypoxia (58.0%) conditions (Supplementary Fig. 1a, Supplementary Data 1). Cumulative fraction analyses showed that hypoxia significantly decreased the interactions of mRNA transcripts with AGO2 (*P* = 2.272E−10, Mann–Whitney U test) (Fig. 1a) and distribution plots further showed that hypoxia attenuated 2100 mRNA transcripts and increased 1528 mRNA transcripts associated with AGO2 (based 2 fold change), respectively (Supplementary Fig. 1b). These results indicate that hypoxia possesses an unrevealed role in suppressing mRNAs loading to AGO2.

MiRNA-targeted mRNAs recruiting to AGO2 for translation repression or decay is mediated through miRISC^2^. To test whether the decreased mRNAs loading to AGO2 is due to the change of miRNA profile under hypoxia, the high-throughput sequencing for miRNA (miRNA-Seq) was conducted, showing that the biogenesis of a small fraction of miRNAs was influenced under hypoxia (Supplementary Fig. 1c, Supplementary Data 2). Similar results were obtained by using other groups’ data of miRNA-seq and microarray in MCF7^31^, miRNA-Seq in 786-O^32^ and murine C166 endothelial^33^ cells (Supplementary Fig. 1d–f). Core catalytic enzymes of the miRNA pathway and some of miRNAs which are even reported regulated by hypoxia^12–14^, were determined to show that the expression levels of these proteins (DICER, Drosha, AGO2 and TARBP2) were inconsistently and slightly changed in 7 different cell lines under hypoxia (Supplementary Fig. 1g). In response to hypoxia, EGFR interacts and phosphorylates AGO2 at the position of Tyr393, which inhibits the interaction of AGO2 with DICER to modulate parts of long-loop structure pre-miRNAs processing^12^, such as pre-miR-192, pre-miR-31 and pre-miR-193a, but not of the short-loop structure pre-miR-21. Consistent with this, the expression of let-7a, miR-21 and miR-19b were not changed under hypoxic (Supplementary Fig. 1h). These results illustrate that hypoxia stress has a prior effect on the biogenesis of only some special miRNAs, but which is dependent on cell types.

The Northern blot and qRT-PCR results showed that hypoxia had no effect on let-7a biogenesis (Supplementary Fig. 1h, 2a). Furthermore, the RIP-Northern blot assay revealed that hypoxia had little role in regulating let-7a loading to AGO2 in HeLa (Fig. 1b), 293T (Supplementary Fig. 2b) and H1299 cell lines (Supplementary Fig. 2c). However, the mRNA of *c-MYC*, targeted by let-7a, binding to AGO2 was decreased under hypoxia stress (Fig. 1b; Supplementary Fig. 2b). We reconstructed a green fluorescent protein (GFP) reporter as the let-7a target, in which four-repeated let-7a binding site sequences were rebuilt into the 3′-untranslated region (3′-UTR) of GFP (called GFP-4xlet-7a-BS) (Supplementary Fig. 2d), and stably expressed this reporter in 293T cells, the RIP-qRT-PCR assay showed that hypoxia attenuated *GFP* mRNA loading to AGO2 (Supplementary Fig. 2e). Moreover, we employed an in vitro target mRNA pull-down assay, in which the RNA of HMGA2-3′-UTR mutant contains a characterized native let-7 target site^34^ (Supplementary Fig. 2f), to evaluate the interaction of target mRNA with AGO2 through let-7 under hypoxia stress, and the results showed that hypoxia decreased the association of AGO2 to let-7a-targeted mRNA HMGA2-3′-UTR mutant (Fig. 1c). Thus, we speculate that hypoxia inhibits the miRNA-targeted mRNA loading to AGO2. To further verify this, we analyzed the associations between AGO2 and mRNAs targeted by the top 10 non-difference miRNAs (Supplementary Fig. 2g) under hypoxia condition. Considering that one mRNA might be targeted by several miRNAs, we chose those mRNAs, which are targeted only by top 10 non-difference miRNAs but not by those of hypoxia-reduced miRNAs, for analysis of the RIP-Seq data, and showed that hypoxia significantly attenuated the association between AGO2 and targets with sites of only top 10 non-difference miRNAs (Fig. 1d). Notably, the enriched abundance of mRNA transcript peaks with RIP-Seq data showed that hypoxia attenuated the interaction of *MYC* and *HMGA2* mRNA transcripts with AGO2 (Fig. 1e, f). These results demonstrate that hypoxia decreases miRNA-targeted mRNA loading to AGO2.

### Hypoxia inhibits miRNA-targeted mRNA turnover

To answer whether the suppressed association of miRNA-targeted mRNAs with AGO2 by hypoxia inhibits mRNA decay, the high-throughput sequencing of mRNA (RNA-Seq) were conducted (Supplementary Data 3). Transcript mRNAs FPKM (Fragments per kilobase of exon per million fragments) >1 presented in both samples were analyzed in parallel. The expressions of 1578 targets by the top10 non-difference miRNAs and 1413 non-miRNA targets were identified (mean FPKM > =30 is considered as the effective candidates) and shown as distribution plots (Supplementary Fig. 2h). The results showed that hypoxia significantly increased the abundance of the top 10 non-difference miRNA targets than that of non-miRNA targets (Fig. 1g). These top 10 non-difference miRNA targets were further subdivided into as 6mer, 7mer and 8mer miRNA-seed complementarily paired targets, showing that hypoxia enriched the abundance of miRNA targets with a single 8mer site more than those of miRNA targets with a single 7mer and 6mer site (Fig. 1h). In addition, we employed a let-7 miRISC luciferase reporter assay, in which four-repeated let-7a binding site (BS) sequences were reconstructed into the 3′-UTR of *Renilla* luciferase in the psiCHECK2 vector getting a reporter construct psiCHECK2-4xlet-7a-BS, and the miRISC luciferase activity was normalized by *Firefly* luciferase (Supplementary Fig. 2i). We transfected this reporter into 293T cells, and then treated with hypoxia or CoCl_2_ for indicated times, the results showed that hypoxia stress significantly increased the inhibition of the luciferase reporter activities (Fig. 1i). Furthermore, we transfected let-7a miRNA mimics and negative control into 293T cells stably expressing GFP-4xlet-7a-BS for 24 h, and subsequently treated with hypoxia for 24 h. The GFP protein expression detection by Western blot showed that hypoxia recovered the *GFP* mRNA decay by let-7a mimics (Supplementary Fig. 2j). Meanwhile, a similar experiment of siRNA targeted to endogenous *PTEN* mRNA in HeLa cells was performed, and the results showed that hypoxia similarly recovered the endogenous *PTEN* decay by siRNA (Supplementary Fig. 2k). Moreover, gene ontology (GO) analysis revealed that several distinct gene clusters such as cell division, DNA repair and mRNA processing were gathered under hypoxia (Supplementary Fig. 2l). Taken together, all the above results illustrate that hypoxia impairs miRNA-targeted mRNA loading to AGO2 and thus inhibits mRNA decay.

### Hypoxia promotes the interaction of AGO2 with LUBAC

**Fig. 2:**
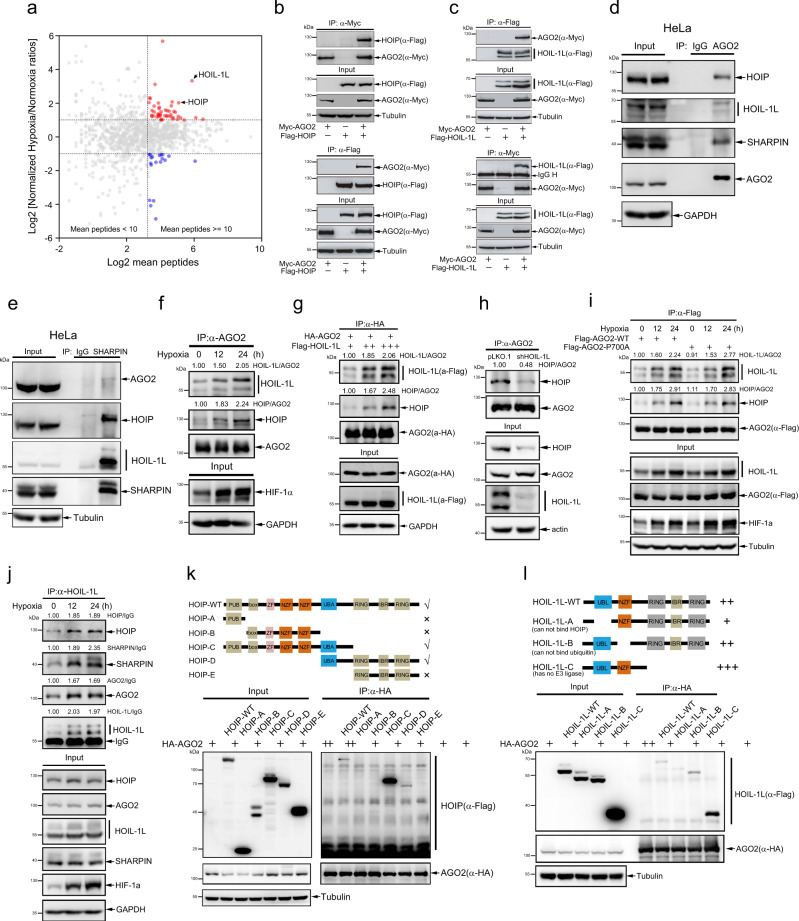
Hypoxia promotes the interaction of AGO2 with LUBAC. **a** Scatter distribution plot of proteins interacting with AGO2 under hypoxia and normoxia conditions were analyzed by mass spectrometry (MS). HeLa cells expressing Flag-AGO2 were treated with hypoxia or normoxia for 24 h, respectively. Cell lysates were used for co-IP with anti-Flag antibody, and followed by MS analysis. **b**, **c** Ectopically expressed Flag-HOIP or Flag-HOIL-1L interacted with AGO2. Myc-AGO2 was co-transfected with Flag-HOIP (**b**) or Flag-HOIL-1L (**c**) into 293T cells, the association of AGO2 with HOIP or HOIL-1L was determined by co-IP/WB. **d** HOIP, HOIL-1L and SHARPIN interacted with AGO2. Lysates from HeLa cells were used by co-IP with anti-AGO2 antibody, and followed by WB. **e** SHARPIN associated strongly with HOIP, HOIL-1L but weakly with AGO2. Lysates form HeLa cells were used by co-IP with anti-SHARPIN antibody, and followed by WB. **f** Hypoxia promoted the interaction of AGO2 with HOIL-1L and HOIP. HeLa cells treated with hypoxia were lysed for co-IP with anti-AGO2 antibody, and followed by WB with anti-HOIP and anti-HOIL-1L antibodies. **g** Increasing expression of HOIL-1L promoted AGO2 recruiting more LUBAC. Flag-HOIL-1L was transfected in a dose-increasing manner with HA-AGO2 into 293T cells. co-IP was performed with anti-HA antibody, and then endogenous HOIP and Flag-HOIL-1L bound to AGO2 were conducted by WB. **h** Knockdown of HOIL-1L decreased the interaction of AGO2 with HOIP. Stable HOIL-1L-knockdown HeLa cells were used for co-IP/WB with indicated antibodies. **i** AGO2 prolyl-4-hydroxylation did not influence its interaction with HOIL-1L and HOIP. 293T cells transfected with Flag-AGO2-WT or prolyl-4-hydroxylation mutant Flag-AGO2-P700A were treated with hypoxia for indicated times, and then lysed for co-IP/WB with indicated antibodies. **j** Hypoxia enhanced the association of HOIL-1L with HOIP and AGO2. HeLa cells treated with hypoxia for indicated times were lysed for co-IP/WB with indicated antibodies. **k**, **l** Analyses of HOIP and HOIL-1L binding domains with AGO2. Various truncated forms of Flag-HOIP (**k**) or Flag-HOIL-1L (**l**) were individually co-transfected with HA-AGO2 into 293T cells, and then lysates were used by co-IP with anti-HA antibody, followed by WB.

To investigate the underlying molecular mechanism, we analyzed proteins interacting with AGO2 in HeLa cells expressing Flag-AGO2 under hypoxia for 24 h by the mass spectrometry (MS). The interactions of AGO2 with two proteins HOIL-1L and HOIP, the components of LUBAC^16–19^, were identified, which were augmented by hypoxia (Fig. 2a, Supplementary Data 4). To validate whether AGO2 interacts with the components of LUBAC, lysates from 293T cells transfected with Myc-AGO2 and Flag-HOIP (Fig. 2b) or Flag-HOIL-1L (Fig. 2c) were used for co-immunoprecipitation (co-IP) with anti-Flag or anti-Myc antibody, respectively. The following immunoblotting results showed that AGO2 indeed interacted with HOIP and HOIL-1L (Fig. 2b, c). More convincingly, the association of endogenous AGO2 with HOIP, HOIL-1L and SHARPIN in HeLa cells were determined by co-IP with anti-AGO2 antibody (Fig. 2d). Since SHARPIN is also one of the components of LUBAC so we next confirmed the possibility of SHARPIN binding with AGO2. HeLa cell lysates were used for co-IP with anti-SHARPIN antibody, and the result showed that both HOIP and HOIL-1L were strongly but AGO2 weakly pulled down by SHARPIN (Fig. 2e). Taken together, all the above results illustrate that AGO2 interacts with LUBAC.

More importantly, hypoxia greatly increased the interaction of endogenous of AGO2 with HOIL-1L and HOIP (Fig. 2f). To uncover why hypoxia augmented the interaction of LUBAC with AGO2, we investigated the expression level of LUBAC components in HeLa cells under hypoxia or in 293T cells treated with CoCl_2_, and found that hypoxia induced only HOIL-1L expression (Supplementary Fig. 3a), which is consistent with reported previously^35,36^. Further, we confirmed that hypoxia induced the mRNA transcription of *HOIL-1L* but not of *HOIP* and *SHARPIN* by qRT-PCR (Supplementary Fig. 3b). Next to examine whether HIF-1α is the transcript factor of *HOIL-1L*, the *HOIL-1L* promoter was subcloned to the luciferase reporter pGL3-basic to get an *HOIL-1L* luciferase reporter, which was transfected into 293T cell for 24 h, subsequently treated with hypoxia or CoCl_2_ for the indicated times. The luciferase reporter results showed that hypoxia increased the *HOIL-1L* promoter activity (Supplementary Fig. 3c, d). Collectively, our results demonstrated that hypoxia induces the expression of HOIL-1L and thus increases the interaction of LUBAC with AGO2, which is consistent with the finding that deficiency of HOIL-1L lead to drastically destabilization of the LUBAC complex^16–19^.

To support that the increased association of HOIP with AGO2 is due to the more expression of HOIL-1L under hypoxia, Flag-HOIL-1L in a dose-increasing manner was transfected with the same amount of HA-AGO2 into 293T cells to mimic hypoxia condition. The co-IP/WB result showed that AGO2 recruited more endogenous HOIP along with increasing expression of HOIL-1L (Fig. 2g). On the contrary, knockdown of HOIL-1L enormously decreased the interaction of HOIP with AGO2 in HeLa cells (Fig. 2h). Since AGO2 is prolyl-4-hydroxylated at the residue proline 700 by P4H-α and P4H-β^37^, we investigated whether AGO2 prolyl-4-hydroxylation influences its interaction with LUBAC. 293T cells transfected with Flag-AGO2-WT or prolyl-4-hydroxylation mutant Flag-AGO2-P700A were treated with hypoxia for indicated times, cell lysates were used for co-IP with anti-Flag antibody. The results showed that hypoxia increased the interactions of AGO2-P700A with HOIP and HOIL-1L, as a similar pattern with AGO2-WT, indicating the prolyl-4-hydroxylation of AGO2 at P700 did not influence its interaction with HOIP and HOIL-1L under hypoxia stress (Fig. 2i). Moreover, to explore whether the induced HOIL-1L by hypoxia recruits more AGO2, HOIP or/and SHARPIN, HeLa cells treated with hypoxia for indicated times were lysed for co-IP with anti-HOIL-1L antibody, and then followed by Western blotting, which showed that hypoxia increased the association of HOIL-1L with HOIP, SHARPIN and AGO2 (Fig. 2j). We further examined the domains of HOIP or HOIL-1L that are involved in their protein-protein interaction with AGO2, Flag-tagged full length of HOIP or HOIL-1L and their domain deletion/truncated forms were co-transfected with HA-tagged AGO2 into 293T cells, then we used the anti-HA co-IP/WB to show that UBA domain of HOIP and UBL domain of HOIL-1L take the responsibility for the association with AGO2 (Fig. 2k, l), respectively. Taken together, all the above results illustrate that AGO2 interacts with LUBAC.

### M1-linked linear ubiquitination of AGO2 is mediated by LUBAC in cells

LUBAC is the only enzyme which catalyzes the formation of linear poly-ubiquitin chain to its substrates, such as NEMO, RIPK1^17^, RIPK2^38^, ASC^39^ and STAT1^40^ in the regulation of inflammation and immune signaling, Htt-polyQ aggregates related to neurodegenerative disease^41^ and CENP-E for chromosome alignment^42^. Recently, hundreds of linear polyubiquitin-modified substrates were identified through the mass spectrometry analysis combined with lysine-less internally tagged ubiquitin strategy^30^, indicating that M1-Ubi might participate in various cellular physiological processes. Such being in the case of AGO2 directly interacting with LUBAC which we have demonstrated above, we raised a question whether AGO2 is M1-linked linear poly-ubiquitinated. Firstly, we generated a mutant ubiquitin-7KR through mutation of all seven lysines of ubiquitin to arginines (Supplementary Fig. 4a), so that the formation of poly-ubiquitin chain is a unique form of linear poly-ubiquitination linked by the first methionine (Met^1^) rather than by lysine residues. Then we co-transfected AGO2 with or without ubiquitin-7KR into 293T cells, through IP with anti-Flag antibody and then immunoblotting by a specific linear poly-ubiquitin antibody LUB9 clone (or IE3 clone), and found that AGO2 was linear-ubiquitinated and this was increased by ectopically expressing ubiquitin-7KR (Supplementary Fig. 4b). In addition, a Met^1^-linkage-specific ubiquitin binder (M1-SUB)^43^ pull-down assay also showed a similar result (Supplementary Fig. 4c).

**Fig. 3:**
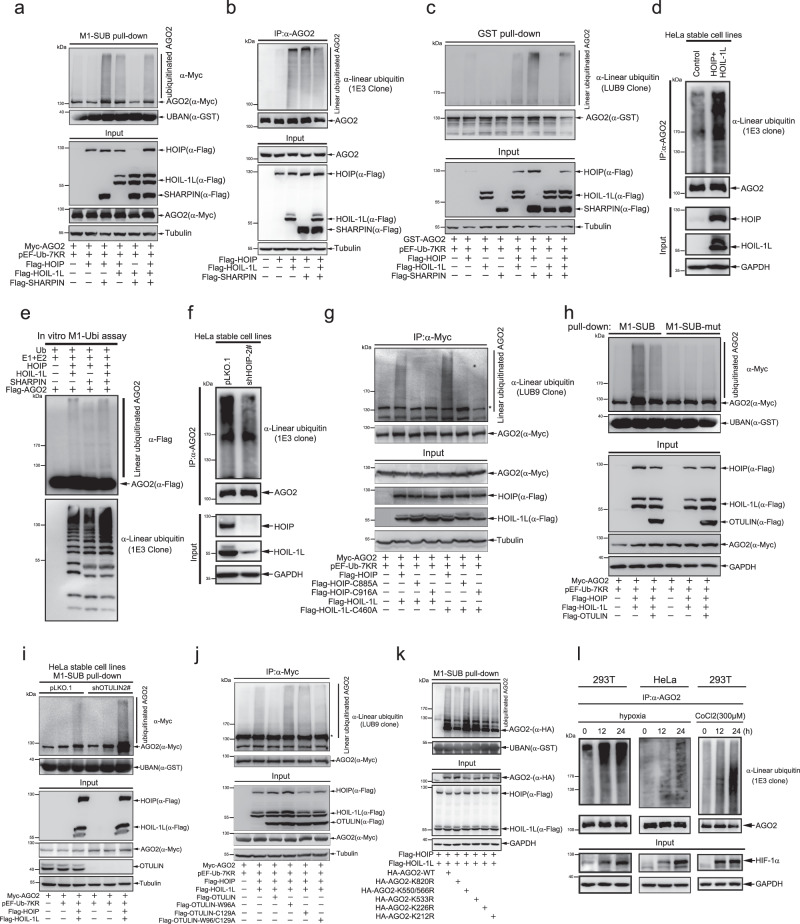
M1-Ubi of AGO2 is catalyzed by LUBAC, which is induced by hypoxia. **a** LUBAC catalyzed M1-Ubi of AGO2. LUBAC related plasmids were transfected into 293T cells and subsequently performed by M1-SUB pull-down assay for detection of AGO2 M1-Ubi. **b** M1-Ubi of endogenous AGO2 was enhanced by LUBAC. 293T cells were co-transfected with components of LUBAC. Lysates were used for IP with anti-AGO2 antibody, followed by WB with a specific linear ubiquitin antibody 1E3 clone. **c** LUBAC catalyzed M1-Ubi of GST-AGO2 in vitro. Purified GST-AGO2 were incubated with lysates from 293T cells transfected with components of LUBAC, and followed by GST pull-down/WB assay with a specific linear ubiquitin antibody LUB9 clone. **d** LUBAC increased M1-Ubi of endogenous AGO2. Stable HeLa cells expressing HOIP and HOIL-1L were lysed for IP with anti-AGO2 antibody, and then determined M1-Ubi of AGO2 by WB with antibody 1E3 clone. **e** In vitro M1-Ubi assay for AGO2. Flag-AGO2 purified from 293T cells was co-incubated with purified ubiquitin and LUBAC proteins for in vitro M1-Ubi assay. **f** Knockdown of HOIP inhibited AGO2 M1-Ubi. Lysates from stable HeLa-shHOIP or -pLKO.1 cells were used for IP/WB. **g** AGO2 M1-Ubi was dependent on the catalytic activity of HOIP, but not that of HOIL-1L. 293T cells were co-transfected with indicated plasmids, and M1-Ubi of AGO2 was detected by IP/WB. **h** M1-Ubi of AGO2 was removed by OTULIN. 293T cells were co-transfected with indicated plasmids, and M1-Ubi of AGO2 was analyzed by M1-SUB pull-down/WB. **i** Knockdown of OTULIN increased AGO2 M1-Ubi. Stable HeLa-shOTULIN cells were transfected with indicated plasmids, and AGO2 M1-Ubi was analyzed by M1-SUB pull-down/WB. **j** OTULIN mutants (W96A, C129A) did not inhibit AGO2 M1-Ubi. OTULIN or indicated OTULIN mutants were transfected into 293T cells, respectively. M1-Ubi of AGO2 was analyzed by IP/WB. **k** K820 is a major site for M1-Ubi of AGO2. The M1-Ubi of AGO2-WT and indicated AGO2 mutants were detected by M1-SUB pull-down and WB. **l** Hypoxia-induced M1-Ubi of AGO2. 293T and HeLa cells were treated with hypoxia or CoCl_2_ (300 μM) for indicated times, and AGO2 M1-Ubi was determined by IP/WB.

Since AGO2 interacted with LUBAC, so we speculated that LUBAC is the E3 ligase of AGO2 for M1-Ubi. In order to confirm this, the components of LUBAC with (Fig. 3a) or without (Fig. 3b) myc-AGO2 were co-transfected into 293T cells, respectively. The M1-SUB pull-down assay find that LUBAC increased M1-Ubi of exogenous AGO2 (Fig. 3a). Meanwhile, M1-Ubi of endogenous AGO2 was also enhanced by LUBAC (Fig. 3b). To further support this, lysates from 293T cells ectopically expressing LUBAC complexes were co-incubated with purified GST-AGO2, subsequently pulled down by GST beads and immunoblotted by anti-LUB9, showing that LUBAC promoted M1-Ubi of AGO2 (Fig. 3c). Besides, M1-Ubi of endogenous AGO2 was augmented when HOIP and HOIL-1L were stably overexpressed in HeLa cells (Fig. 3d). More convincingly, we investigated LUBAC mediating M1-Ubi of AGO2 in vitro, and showed that LUBAC was capable of catalyzing M1-Ubi of AGO2 in vitro (Fig. 3e). These results demonstrate that AGO2 is a linear poly-ubiquitination substrate of LUBAC E3 ligases. Considering that SHARPIN is one of the essential components of LUBAC, we further investigated whether SHARPIN is necessary for M1-Ubi of AGO2. 293T cells stably knocking down SHARPIN were co-transfected AGO2 with HOIP and HOIL-1L, the M1-SUB pull-down assay showed that knockdown of SHARPIN slightly decreased M1-Ubi of AGO2 mediated by ectopic expression of HOIP and HOIL-1L (Supplementary Fig. 4d). HOIP is the most crucial component of LUBAC, which catalyzes the formation of linear ubiquitin chain by its RING-IBR-RING (RBR) and extension LDD domains^44^. Stable knockdown of HOIP by shRNA in HeLa cells dramatically decreased the M1-Ubi of AGO2 (Fig. 3f). The sites of the cysteine at 885 in RING-IBR-RING (RBR) domain and the cysteine at 916 in the LDD domain are essential for HOIP enzymatic activity^44^, and the cysteine 460 is the key site for HOIL-1L E3 ligase activity. We co-transfected inactive mutant HOIP-C885A, HOIP-C916A or HOIL-1L-C460A with AGO2 into 293T cells for IP (Fig. 3g) and M1-SUB pull-down (Supplementary Fig. 4e), the results revealed that M1-Ubi of AGO2 was dependent on the enzymatic activity sites C885 and C916 of HOIP but not C460 of HOIL-1L.

The RBR region of HOIP is sufficient to generate linear poly-ubiquitin chain in vitro whereas the full-length HOIP cannot, which is due to that the catalytic activity of HOIP is self-inhibited but can be released upon the LUBAC formation of HOIP with HOIL-1L, SHARPIN or both of them^45^. Gliotoxin (a fungal metabolite), which can block the enzymatic activity of LUBAC by selective binding to the RBR region of HOIP^46^, strongly decreased AGO2 M1-Ubi within half of 1 h and more apparently in 1 h (Supplementary Fig. 4f). We also investigated the functional domain of HOIL-1L in mediating AGO2 M1-Ubi. Ubiquitin-like domain (UBL) of HOIL-1L is required for binding to HOIP to achieve the enzymatic activity, Npl4 type-zinc-finger domain (NZF) of HOIL-1L is required for binding to linear ubiquitin chain and RBR of HOIL-1L possesses E3 ubiquitin ligase. We co-transfected the functional domain truncated/deleted forms of HOIL-1L with HOIP and AGO2 into 293T cells for M1-SUB pull-down assay, the results showed that deletion of UBL domain other than NZF and RBR domains of HOIL-1L could not reshape HOIP catalytic activity to induce M1-Ubi of AGO2 (Supplementary Fig. 4g). Collectively, these above results demonstrate that LUBAC catalyzes M1-Ubi of AGO2.

### OTULIN is a de-linear-ubiquitinase of AGO2

It has been known that OTULIN, CYLD, and A20 negatively regulate linear ubiquitin chain assembly via different mechanisms. OTULIN antagonizes LUBAC signaling highly specific hydrolyzing linear poly-ubiquitin chain^20,21,38^. CYLD is not only as a de-linear-ubiquitinase to remove linear poly-ubiquitin chain but also cleaving K63-linked poly-ubiquitin chain^23^. The C-terminal zinc-finger 7 (ZF7) domain of A20 can directly bind to linear ubiquitin chain and impairs the interaction of LUBAC with substrates (such as NEMO) for M1-Ubi^22^. Considering that the highly specific de-linear-ubiquitin characteristic of OTULIN, we further investigated whether OTULIN is a de-linear-ubiquitinase of AGO2. HOIP, HOIL-1L and AGO2 were co-transfected with or without OTULIN into 293T cells for IP and M1-SUB pull-down assay, the results showed that OTULIN greatly reduced M1-Ubi of AGO2 mediated by LUBAC (Fig. 3h; Supplementary Fig. 4h). In contrast, knockdown of OTULIN by shRNA or siRNA enormously strengthened AGO2 M1-Ubi (Fig. 3i; Supplementary Fig. 4i, j). Moreover, M1-Ubi of AGO2 was removed by wild-type OTULIN but not mutants, the catalytic cysteine (C129A) and the tryptophan residue (W96A), which both are involved in linear-ubiquitin chain binding^20^ (Fig. 3j; Supplementary Fig. 4k).

OTULIN cleaves linear ubiquitin chains via its PIM motif binding with the N-terminal PNGase/UBA (PUB) domain of HOIP. We generated HOIP mutants including N85/Y94A, Y82A, N102D, Y82A/N102D, which all interferes the association of HOIP with OTULIN. The M1-SUB pull-down assay showed that these HOIP mutants greatly promoted AGO2 M1-Ubi than that of wild-type HOIP (Supplementary Fig. 4l). However, a relative lower expression level of OTULIN was enough to cleave the linear ubiquitin chain of AGO2, even such an augment of AGO2 M1-Ubi increased by HOIP-N85/Y94A, and this effect was not increased by the higher level of OTULIN (Supplementary Fig. 4m), which suggested that HOIP N85/Y94A mutant was not able to totally block the interaction of HOIP with OTULIN and OTULIN possessed a powerful role in de-linear-ubiquitination of AGO2. Moreover, since Y56 of OTULIN is conserved and critical for interacting with the PUB domain of HOIP and its phosphorylation negatively regulates this interaction^47^, we generated a mutant OTULIN-Y56F to abolish the phosphorylation for increasing the association of OTULIN with HOIP, which expectedly enhances de-linear-ubiquitination by OTULIN. However, de-linear-ubiquitination by wild-type OTULIN was not blocked by the addition of OTULIN-Y56F, which suggested a small amount of wild-type OTULIN was enough to cleave the linear ubiquitin chains on AGO2 (Supplementary Fig. 4n). Thus, the above results demonstrate that OTULIN is a de-linear-ubiquitinase of AGO2.

### K820 is one of M1-Ubi sites of AGO2 and hypoxia induces M1-Ubi of AGO2 catalyzed by LUBAC

To further identify M1-Ubi sites of AGO2, HeLa cells were treated with hypoxia for 12 or 24 h and then lysed in denaturing lysis buffer, which avoided pulldown of ubiquitin chains associated (not covalently modified) in IP with anti-AGO2 antibody. The IP-WB results showed that hypoxia strongly enhanced AGO2 M1-Ubi (Supplementary Fig. 5a) and the IP-MS analysis revealed that 19 lysine residues of AGO2 were ubiquitinated under hypoxic condition (Supplementary Fig. 5b). Moreover, lysates from 293T cells transfected with HA-AGO2, Flag-HOIP and Flag-HOIL-1L were also used for IP-MS analysis, revealing that AGO2 was multiply linear-ubiquitinated at 19 lysines (Supplementary Fig. 5b). 12 lysine residues of AGO2 ubiquitination were overlapped among these residues identified as AGO2 ubiquitination sites under hypoxic condition and transiently transfection with Flag-HOIP and Flag-HOIL-1L by MS analysis (Supplementary Fig. 5b, c, Supplementary Data 5). We generated AGO2 functional domain constructs to probe its possible M1-Ubi sites, M1-SUB pull-down assay showed that M1-Ubi sites were mainly distributed in small RNA binding domains PAZ and MID as well as nuclease activity domain PIWI of AGO2 (Supplementary Fig. 5d, e), implying that AGO2 was linear-ubiquitinated at multiple lysines. We have generated point or combined mutations for these lysines into arginines, and found that all those mutations could not completely abolish but the single-mutation K820R could reduce about a half of AGO2 M1-Ubi compared to that of AGO2-WT (Fig. 3k; Supplementary Fig. 5f). Most importantly, considering that hypoxia induced the association of AGO2 with LUBAC (Fig. 2), we detected the dynamics of AGO2 M1-Ubi under hypoxia condition, showing that both hypoxia and CoCl_2_ strongly enhanced AGO2 M1-Ubi (Fig. 3l). Taken together, these above results demonstrate that hypoxia induces M1-Ubi of AGO2 catalyzed by LUBAC.

### HOIP and HOIL-1L are co-localized with miRISC

**Fig. 4:**
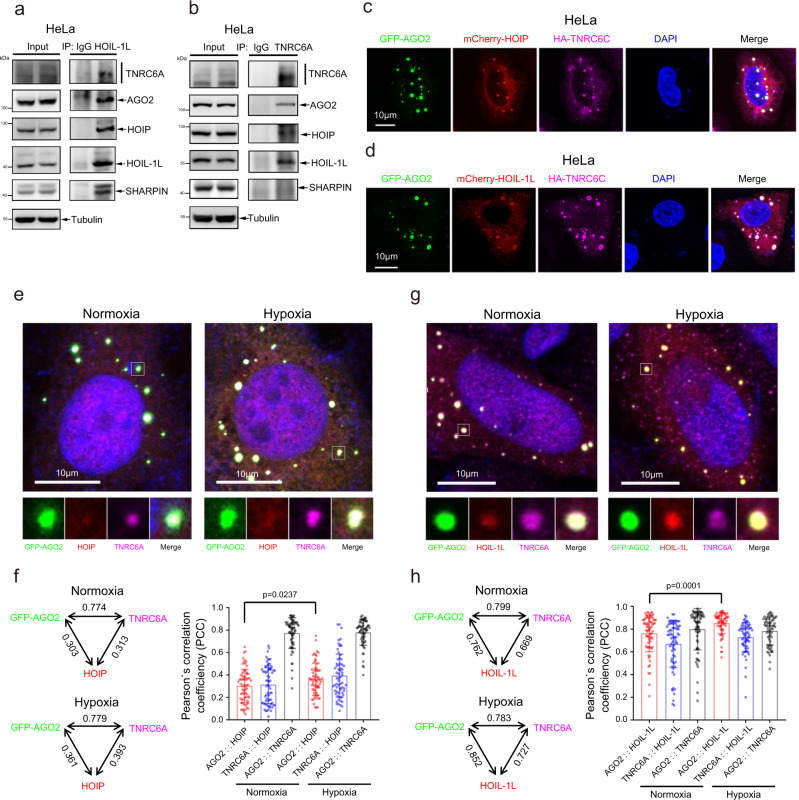
HOIP and HOIL-1L co-localize with miRISC. **a**, **b** AGO2/TNRC6A interacted with LUBAC. HeLa cells were lysed for co-IP with anti-HOIL-1L (**a**) or anti-TNRC6A (**b**) antibody, and then the interactions between AGO2/TNRC6A with HOIP and HOIL-1L were detected by WB with indicated antibodies. **c**, **d** LUBAC was co-localized with AGO2/TNRC6C of miRISC. GFP-AGO2 and HA-TNRC6C were co-transfected with mCherry-HOIP (**c**) or mCherry-HOIL-1L (**d**) into HeLa cells, the co-localization of GFP-AGO2 (green), TNRC6C (purple) with mCherry-HOIP (red) or mCherry-HOIL-1L (red) were observed by immunofluorescence staining. **e**–**h** Hypoxia increased the co-localization of HOIP and HOIL-1L to AGO2/TNRC6A miRISC. GFP-AGO2 was transfected into HeLa cells, the co-localization of GFP-AGO2 (green), TNRC6A (purple) with HOIP (red) or HOIL-1L (red) were observed by immunofluorescence staining. The scale of the magnified region (white frame) is 2 μm x 2 μm (**e**, **g**). Co-localization between GFP-AGO2/TNRC6A with HOIP or HOIL-1L were analyzed by Pearson’s correlation coefficient. Data were mean ± s.d., and *P*-values were determined by unpaired two-sided t-test (**f**, **h**).

MiRNA assembles with AGO2 and TNRC6/GW182 into miRNA-induced silencing complex (miRISC) to directly prompt post-transcriptional silencing of targeted mRNAs. To investigate whether HOIP and HOIL-1L are recruited to miRISC, HeLa cell lysates were used for co-IP with anti-HOIL-1L antibody. The result showed that not only HOIP, SHARPIN and AGO2 but also TNRC6A/GW182 bound to HOIL-1L (Fig. 4a). Reciprocally, HOIP, HOIL-1L and AGO2 were pulled down by co-IP with anti-TNRC6A antibody (Fig. 4b). Furthermore, we co-transfected GFP-AGO2, HA-TNRC6C with mCherry-HOIP or mCherry-HOIL-1L into HeLa cells, the fluorescent immunostaining showed that HOIP and HOIL-1L were co-localized with the representative foci structure of AGO2/TNRC6C miRISC (Fig. 4c, d). To explore more co-localization details of HOIP and HOIL-1L with miRISC under hypoxic condition, HeLa cells transfected with GFP-AGO2 were separately treated with normoxia and hypoxia for 24 h, endogenous HOIP or HOIL-1L was found in punctate foci structures that partially co-localized with GFP-AGO2/TNRC6A miRISC under both normoxia and hypoxia conditions by immunofluorescent staining, and pearson’s correlation coefficients (PCC) of co-localization of AGO2::HOIP (Fig. 4e, f) and AGO2::HOIL-1L (Fig. 4g, h) under hypoxia were more than those of in normoxia condition. Taken together, all the above results demonstrate that HOIP and HOIL-1L co-localize to miRISC, and the interaction of miRISC with HOIL-1L is much more than that of with HOIP.

AGO2 interacts with TNRC6/GW182 for miRISC assemble is promoted by phase separation^48^. Indeed, we found the cytoplasmic concentrating foci of miRISC formed by GFP-AGO2 and TNRC6A in HeLa cells (Supplementary Fig. 6a). Moreover, the living HeLa cells showed a fusion of two adjacent GFP-AGO2 punctate foci after transient transfection of GFP-AGO2 (Supplementary Fig. 6b). Fluorescence recovery after photobleaching (FRAP) experiments displayed that plenty of the GFP-AGO2 molecules in punctate foci were quickly exchanged in the cytoplasm, and approximately 40% of GFP-AGO2 punctate foci fluorescence were recovered within 16 min (Supplementary Fig. 6c, d). Next, we wandered whether M1-Ubi of AGO2 induced by LUBAC regulates the processing of miRISC phase separation. HeLa cells stably overexpressing HOIP and HOIL-1L or knocking down HOIP were employed to obtain the high level and low level M1-Ubi of AGO2, respectively. The co-IP results showed that the AGO2 M1-Ubi levels did not influence the interaction of AGO2 with TNRC6A (Supplementary Fig. 6e, f). Consistently, the number of GFP-AGO2/TNRC6A co-localization foci per cell was not influenced when HOIP and HOIL-1L were stably overexpressed or OTULIN was knocked down by shRNA in HeLa cells (Supplementary Fig. 6g, h). Conversely, decreased AGO2 M1-Ubi through knocking down of HOIP by shRNA in HeLa cells did not affect the number of GFP-AGO2/HA-TNRC6C co-localization foci per cell (Supplementary Fig. 6i). These results illustrate that M1-Ubi of AGO2 is not involved in the processing of miRISC phase separation. Taken together, all the above results demonstrate that HOIP and HOIL-1L are co-localized with miRISC.

### M1-Ubi of AGO2 impairs the miRISC activity

**Fig. 5:**
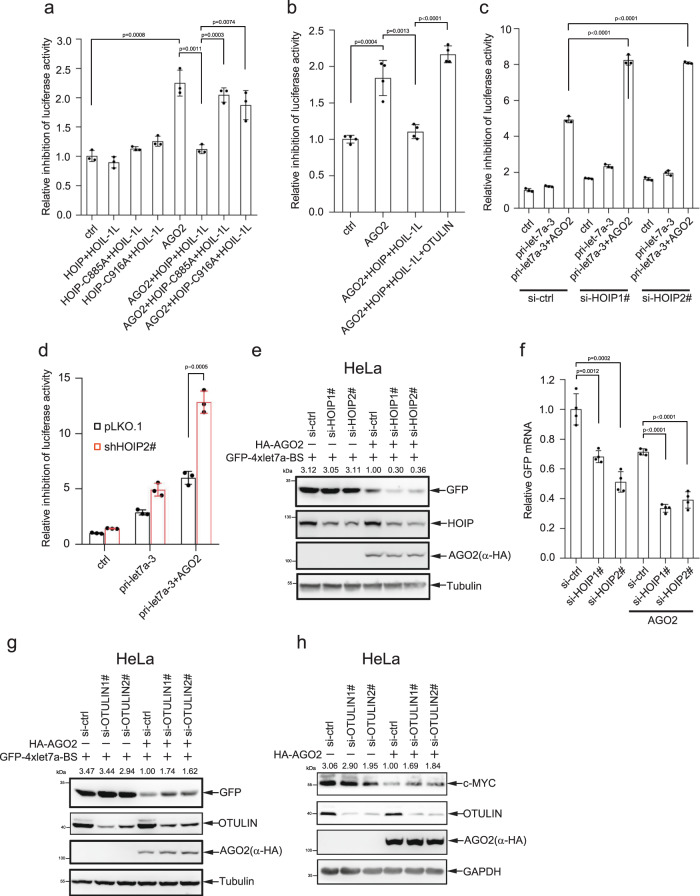
M1-Ubi of AGO2 inhibits the miRISC activity. **a** HOIP/HOIL-1L, but not HOIP-C885A/HOIL-1L and HOIP-C916A/HOIL-1L reduced the inhibition of luciferase activity mediated by AGO2. **b** OTULIN recovered the reduced inhibition of luciferase activity by LUBAC. **c**, **d** Knockdown HOIP by siRNAs in 293T cell (**c**) or by shRNA in HeLa cell (**d**) increased the inhibition of luciferase activity mediated by AGO2. 293T cells (**a**–**c**) or HeLa-shHOIP cells (**d**) transfected with indicated plasmids were harvested for the psiCHECK2-4xlet-7a-BS of dual-luciferase assay. Dual-luciferase reporter assay data were mean ± s.e.m., *n* = 3 (**a**, **c**, **d**) or *n* = 4 (**b**) biologically independent experiments, and *P*-values were determined by unpaired two-sided t-test. **e**, **f** Knockdown of HOIP increased let-7a-targeted GFP protein and mRNA levels. HeLa cells were transfected with indicated plasmids and siRNAs, the GFP protein expression was detected by WB (**e**). 293T cell stably expressing GFP-4x-let-7a-BS was transfected with HOIP siRNAs, and then total RNAs were extracted for detection of *GFP* mRNA levels by qRT-PCR. Data were mean ± s.d., *n* = 4 biologically independent samples, and *P*-values were determined by unpaired two-sided t-test (**f**). **g**, **h** Knockdown of OTULIN decreased let-7a-targeted *GFP* and *c-MYC* decay. HeLa cells were transfected with indicated plasmids and siRNAs, the proteins GFP (**g**) and c-MYC (**h**) expression were measured by WB analysis.

To identify whether M1-Ubi of AGO2 influences miRNA-guided gene silencing, we co-transfected the reporter psiCHECK2-4xlet-7a-BS, HOIP/HOIL-1L or HOIP-mutants/HOIL-1L with or without Myc-AGO2 into 293T cells. The results showed that AGO2 significantly increased the inhibition of the luciferase reporter activities, which could be significantly reduced by HOIP/HOIL-1L but not by HOIP-C885A/HOIL-1L and HOIP-C916A/HOIL-1L (Fig. 5a), suggesting that M1-Ubi of AGO2 might repress miRNA-guided gene silencing. To further verify this, we co-transfected the psiCHECK2-4xlet7a-BS, AGO2, HOIP and HOIL-1L with or without OTULIN into 293T cells, and showed that OTULIN antagonized the decreased inhibition of the luciferase activities by LUBAC (Fig. 5b). Moreover, we co-transfected psiCHECK2-4xlet7a-BS reporter, pri-let-7a-3, AGO2 with HOIP siRNAs and found that knockdown of HOIP greatly increased the inhibition of the luciferase reporter activities mediated by AGO2 (Fig. 5c). Similarly, stable knockdown of HOIP by shRNA in HeLa cells also enhanced the inhibition of the luciferase reporter activities increased by AGO2 (Fig. 5d).

To further support this, we co-transfected GFP-4xlet-7a-BS, HOIP siRNA with or without HA-AGO2 into HeLa cells. As expectedly, the GFP expression level was suppressed AGO2, and this effect was much stronger when HOIP was knocked down by siRNA (Fig. 5e). Due to AGO2/miRNA-mediating target gene silencing on the post-transcript level, so we investigated the mRNA level measured by QRT-PCR in 293T cells stably expressing GFP-4xlet-7a-BS which was co-transfected with AGO2 and HOIP siRNAs, to show that knockdown of HOIP increased the let-7a miRISC activity for *GFP* mRNA decay (Fig. 5f). In contrast, knockdown of OTULIN by siRNAs suppressed the let-7 miRISC activities for silencing of targeted GFP (Fig. 5g) and endogenous c-MYC (Fig. 5h). Taken together, these data demonstrate that M1-Ubi of AGO2 undermines miRNA-mediated gene silencing.

### M1-Ubi of AGO2 does not influence miRNA processing

Next, we tried to explore the mechanism underlying M1-Ubi of AGO2 affecting the miRNA pathway. Knockdown of either HOIP or OTULIN to reshape the status of M1-Ubi of AGO2 did not affect the expression levels of core proteins in the miRNA pathway, such as DICER, AGO2 and TARBP2 (Supplementary Fig. 7a, b). Further, Myc-AGO2 and Flag-HA-DICER were co-transfected into HeLa cells in which HOIP was knocked down by shRNA, the co-IP/WB results showed that knockdown of HOIP does not influenced the interaction of AGO2 with DICER (Supplementary Fig. 7c). Moreover, AGO2 was ectopically expressed with HOIL-1L, HOIP or HOIP-mutants in 293T cells to show that the association of AGO2 with endogenous DICER was not affected by overexpression of either HOIP/HOIL-1L or HOIP-C885A/HOIL-1L and HOIP-C916A/HOIL-1L mutants (Supplementary Fig. 7d). These results demonstrate that M1-Ubi of AGO2 has no role in regulating the expression level of core proteins in the miRNA pathway and the binding of AGO2 with DICER.

To estimate whether M1-Ubi of AGO2 participates in the pre-miRNA processing, we employed an in vitro pre-miRNA processing assay. Lysates from 293T cells transfected with indicated plasmids were immunoprecipitated by HA-AGO2, then the beads were co-incubated with purified biotin-tagged pre-let-7a-3 for the following in vitro processing assay. The results revealed that AGO2 effectively promoted the mature let-7a biogenesis, but overexpression of HOIP-WT, -C885A or -C916A and HOIL-1L had no effect on mature let-7a biogenesis processed by AGO2 (Supplementary Fig. 7e), which suggested that M1-Ubi of AGO2 did not influence the pre-let-7a-3 processing. AGO2 possesses the nuclease activity for cleavage of targeted mRNA and passenger strand of small RNA duplexes^3^, which is essential for biogenesis of several miRNAs, such as miR-451^49,50^. Pri-miR-451a and HOIP siRNAs or OTULIN siRNAs were co-transfected with or without AGO2 into 293T cells, and then Northern blotting analysis was performed for assessment of the nuclease activity of AGO2 by the expression level of mature miR-451. As expectedly, AGO2 could remarkably increase the biogenesis of miR-451a; however, it was not influenced by knockdown of either HOIP or OTULIN (Supplementary Fig. 7f, g).

### M1-Ubi of AGO2 impedes its recruitment of miRNA-targeted mRNAs

**Fig. 6:**
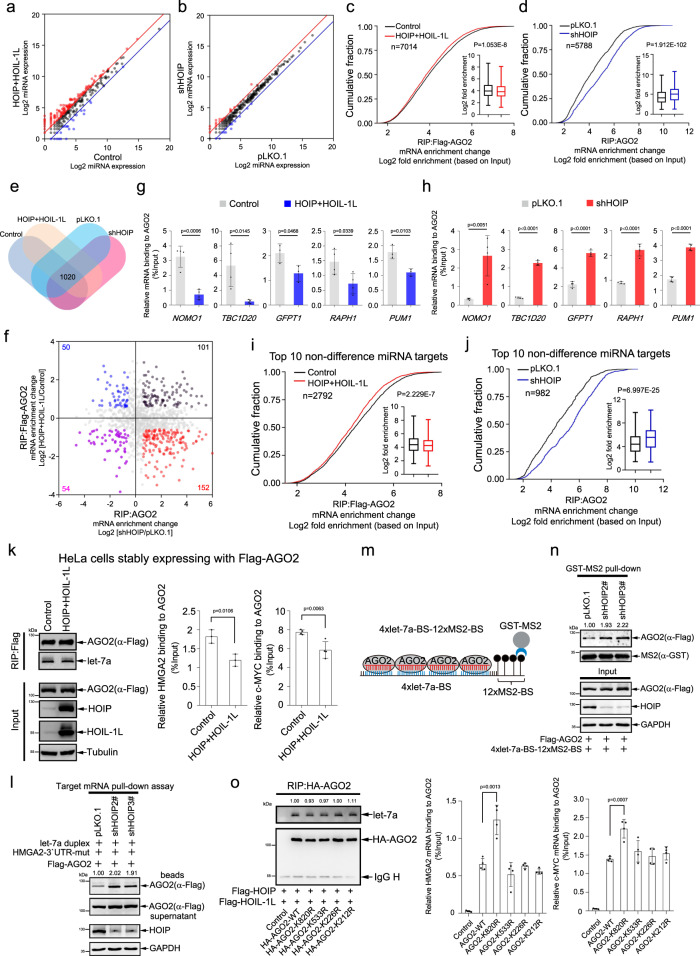
M1-Ubi of AGO2 interferes its recruiting miRNA-targeted mRNAs. **a**, **b** Scatter plots of miRNA expression profiles by miRNA-Seq in stable HeLa-Flag-AGO2 cells expressing HOIP and HOIL-1L (**a**) and HeLa cells knocking down HOIP (**b**). **c**, **d** M1-Ubi of AGO2 inhibited recruitments of mRNAs. Cumulative fraction analyses for mRNA transcripts recruiting to AGO2 were conducted by using RIP-Seq data in stable HeLa-Flag-AGO2 cells expressing HOIP and HOIL-1L (**c**, *n* = 7014 mRNA transcripts) and HeLa cells knocking down HOIP (**d**, *n* = 5788 mRNA transcripts). **e**, **f** Venn diagram analysis (**e**) and scatter plots analysis of mRNA transcripts recruited to AGO2 with more than 1.5-fold changes (**f**). **g**, **h** Several mRNAs bound to AGO2 were examined by RIP/qRT-PCR in above stable HeLa cells. **i**, **j** Cumulative fraction analyses for top 10 non-difference miRNA-targeted mRNA transcripts recruiting to AGO2 (**i**, *n* = 2792 mRNA transcripts; **j**, *n* = 982 mRNA transcripts) were conducted by using RIP-Seq data in stable HeLa cells. **k** LUBAC inhibit the association of let-7-targeted *HMGA2* and *c-MYC* mRNAs with AGO2. *HMGA2* and *c-MYC* mRNAs bound to AGO2 were measured by RIP/qRT-PCR. The RIP efficiency were determined by WB and Northern blotting analysis. **l**–**n** Knockdown of HOIP augmented the associations of let-7a-targeted mRNAs to AGO2. HeLa-shHOIP cells transfected with indicated plasmids were used for target mRNA pull-down assay (**l**) and GST-MS2 pull-down assay (**m**, **n**). **l** RNA bound fraction (beads) and unbound fraction (supernatant) were detected by WB. **m** A schematic illustration of the GST-MS2 pull-down assay. **n** AGO2 associated RNAs through let-7a were assessed by GST-MS2 pull-down. **o** K820R of AGO2 augmented its recruiting with let-7-targeted mRNAs. The abundance of let-7a and let-7a targeted *HMGA2/c-MYC* mRNAs associated with AGO2 or mutants were determined by RIP/northern blotting and RIP/qRT-PCR, respectively. qRT-PCR data were mean ± s.d., *n* > = 3 biologically independent samples, and *P*-values were determined by unpaired two-sided t-test (**g**, **h**, **k**, **o**). In box plots, the lines represent the median, first and third quartiles, the whiskers denote the minima and maxima; *P*-values were calculated using a two-sided Mann–Whitney U test for cumulative fraction analysis (**c**, **d**, **i**, **j**).

We speculated the mechanism for M1-Ubi of AGO2 disrupting the miRISC activity was probably due to the retard of target mRNA recruitment to AGO2. The miRNA profiles by small RNA-Seq in stable HeLa-Flag-AGO2 cells expressing HOIP and HOIL-1L and HeLa cells knocking down HOIP were analyzed to find that a portion of miRNAs were changed (Fig. 6a, b, Supplementary Data 2). Then we performed RIP-Seq with anti-Flag and anti-AGO2 antibody in these two group stable cell lines, respectively. A total of 7014 and 5788 mRNA transcripts associated with AGO2 were identified as overlapping between Control (9595, 73.1%), HOIP + HOIL-1L (9576, 73.2%), pLKO.1 (9060, 63.9%) and shHOIP (12118, 47.8%) cells, respectively (Supplementary Fig. 8a, b). RIP-Seq results showed that overexpression of LUBAC reduced mRNA transcripts bound to AGO2 (*P* = 1.053E−8, Mann–Whitney U test) (Fig. 6c, Supplementary Data 6), whereas knockdown of HOIP significantly increased the interaction of mRNA transcripts with AGO2 (*P* = 1.912E-102, Mann–Whitney U test) (Fig. 6d, Supplementary Data 7). Distribution plots analysis showed that the suppressed AGO2 bound mRNA transcripts (1131, 2-fold up-regulated) were 1.53 fold more than the increased (739, 2-fold down-regulated) by ectopic expression of HOIP and HOIL-1L (Supplementary Fig. 8c). On the contrary, the increased AGO2 bound mRNA transcripts (2221, 2-fold up-regulated) were 2.92 fold more than the reduced (760, 2-fold down-regulated) by knocked down of HOIP (Supplementary Fig. 8d). Combining analysis of above RIP-Seq data displayed 1020 mRNAs associated with AGO2 in all these cell lines (Fig. 6e). Notably, most of these mRNA transcripts bound to AGO2 were significantly reduced in HOIP and HOIL-1L overexpressing cells and simultaneously increased in HOIP knocking down cells (Fig. 6f). To further confirm this, RIP-qRT-PCR was measured to reveal that overexpression of LUBAC significantly decreased and knockdown of HOIP enormously augmented the association of several mRNA transcripts including *NOMO1*, *TBC1D20*, *GFPTA*, *RAPH1* and *PUM1* with AGO2, respectively (Fig. 6g, h).

To make sure that the impaired recruitment of targeted mRNA to M1-Ubi of AGO2 was mediated by miRNA. We analyzed the miRNA expression profile by miRNA-Seq in this two group cell lines (Supplementary Data 2). Comparative analyze to RIP-Seq data, the cumulative fraction results showed that overexpression of HOIP and HOIL-1L significantly inhibited the association of 2792 top-10 non-difference miRNA-targeted mRNAs with AGO2 (Fig. 6i); on the contrary, knockdown of HOIP greatly promoted the recruitment of 982 top-10 non-difference miRNA-targeted mRNAs to AGO2 (Fig. 6j). Consistently, cataloging targets by miRNA-seed complementary sequence showed that overexpression of HOIP and HOIL-1L reduced the interaction of complementarily paired target mRNAs with 6mer (*n* = 2310), 7mer (*n* = 383) and 8mer (*n* = 99) sequences of top-10 non-difference miRNAs to AGO2, respectively (Supplementary Fig. 8e). Conversely, knockdown of HOIP increased the association of complementarily paired target mRNAs with 6mer (*n* = 816), 7mer (*n* = 127) and 8mer (*n* = 39) sequences of top-10 non-difference miRNAs with AGO2, respectively (Supplementary Fig. 8f). These results demonstrate that M1-Ubi of AGO2 impairs its recruitment of miRNA-targeted mRNAs.

To further verify this hypothesis, several biochemistry experiments were conducted. The RIP-northern blotting analysis showed that overexpression of HOIP and HOIL-1L (Fig. 6k) or knockdown of HOIP (Supplementary Fig. 8g) in HeLa cells did not change the association of let-7a with AGO2 and the let-7a biogenesis, but knockdown of HOIP increased *HMGA2* binding to AGO2 while overexpressed of HOIP and HOIL-1L attenuated the association of *HMGA2* and *c-MYC* with AGO2 (Fig. 6k; Supplementary Fig. 8g). Moreover, 293T cells were transfected pri-let-7a-3, AGO2 with HOIP/HOIL-1L or HOIP-C885A/HOIL-1L and HOIP-C916A/HOIL-1L, to show the similar results that M1-Ubi of AGO2 did not affect its recruitment of let-7a as well as the biogenesis of mature let-7a (Supplementary Fig. 8h). However, the RIP-qRT-PCR showed that HOIP/HOIL-1L significantly decreased the binding of let-7a-targeted *HMGA2* and *c-MYC* with AGO2, whereas mutants HOIP-C885A/HOIL-1L and HOIP-C916A/HOIL-1L partly recovered this phenomenon (Supplementary Fig. 8h).

Further, we employed target mRNA pull-down, GST-MS2 pull-down and in vitro target mRNA binding assays to assess the association of let-7a targeted mRNA with AGO2. Four-repeated let-7a binding site sequences followed by twelve-repeated MS2 binding sites sequences were constructed to serve as a let-7a targeted reporter 4xlet-7a-BS-12xMS2-BS (Fig. 6m). HOIP/HOIL-1L, HOIP-C885A/HOIL-1L or HOIP-C916A/HOIL-1L and AGO2 were co-transfected with or without 4xlet-7a-BS-12xMS2-BS into 293T cells or HeLa-shHOIP cells, the target mRNA pull-down, GST-MS2 pull-down and in vitro target mRNA binding assays showed that overexpression of HOIP/HOIL-1L but not of HOIP-C885A/HOIL-1L and HOIP-C916A/HOIL-1L suppressed the interaction of AGO2 with let-7a targeted mRNA HMGA2-3′UTR-mutant and 4xlet-7a-BS-12xMS2-BS (Supplementary Fig. 8i–k), whereas knockdown of HOIP increased the recruitment of let-7a targeted mRNA HMGA2-3′UTR-mutant and 4xlet-7a-BS-12xMS2-BS to AGO2 (Fig. 6l, n). Moreover, we performed a RISC complex assembly assay with native gel, to show that HOIP/HOIL-1L, but not of HOIP-C885A/HOIL-1L and HOIP-C916A/HOIL-1L inhibited the miRISC assembly with targeted mRNA HMGA2-3′UTR-mutant (Supplementary Fig. 8l). Convincingly, the major M1-Ubi site mutation of K820R of AGO2 (Fig. 3k; Supplementary Fig. 5d), significantly increased the interactions of AGO2 with let-7-targeted *HMGA2* and *c-MYC* mRNAs (Fig. 6o). Thus, the above results demonstrate that M1-Ubi of AGO2 impedes miRNA-targeted mRNA loading to AGO2/miRISC.

### M1-Ubi of AGO2 globally promotes mRNA accumulation

**Fig. 7:**
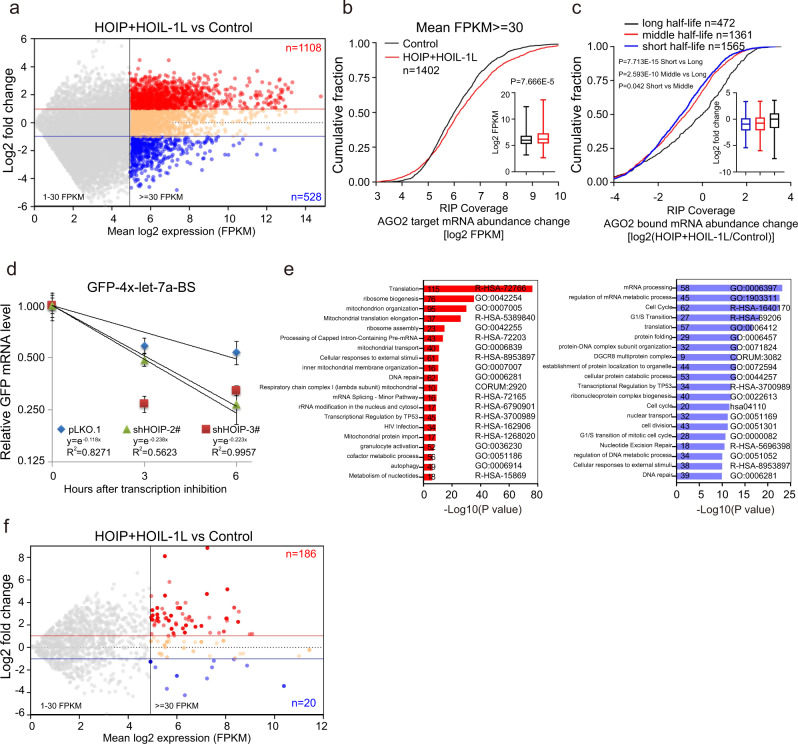
M1-Ubi of AGO2 globally promotes mRNA accumulation. **a**–**c** M1-Ubi of AGO2 promoted mRNA accumulation. Scatter plot of mRNA expression profiles in RNA-Seq (**a**); and cumulative fraction analyses for the abundance of AGO2 bound mRNA transcripts (which were covered by RIP-Seq) in RNA-Seq (**b**, *n* = 1402 mRNAs), which further were categorized as short-, middle- and long-half-life (**c**), in stable HeLa-Flag-AGO2 cells expressing HOIP and HOIL-1L. In box plots, the lines represent the median, first and third quartiles, the whiskers denote the minima and maxima; *P*-values were calculated using a two-sided Mann–Whitney U test for cumulative fraction analysis (**b**, **c**). **d** Knockdown of HOIP accelerated the degradation of let-7a-targeted GFP-4xlet-7a-BS. HeLa cells stably knocking down of HOIP by shRNAs were transfected with GFP-4xlet-7a-BS for 24 h, and then cells were treated with 5 μg/ml Actinomycin D for indicated times. *GFP* mRNA levels were measured by qRT-PCR and normalized with *β-actin* mRNA. Data were mean ± s.d., *n* = 3 biologically independent samples, and *P*-values were determined by unpaired two-sided t-test. **e** Enriched Gene Ontology (GO) categories for genes with 2-fold up-regulated (red, left panel) and down-regulated (blue, right panel) mRNA transcripts when HOIP and HOIL-1L were overexpressed. *P* values for enrichment of the indicated GO category were computed by Metascape. **f** Ectopically expressed LUBAC increased lncRNA transcripts accumulation. Scatter plots of lncRNA expression profiles by RNA-Seq in stable HeLa-Flag-AGO2 cells expressing HOIP and HOIL-1L or control vector.

Next, we asked whether M1-Ubi of AGO2 regulates mRNA accumulation. RNA-Seq was conducted in HeLa-Flag-AGO2 stably expressing the control vector or HOIP/HOIL-1L cell lines to show that the increased mRNA transcripts (1108, FPKM > = 30) were 2.10 fold more than the decreased (528, FPKM > = 30) by overexpression of HOIP/HOIL-1L (Fig. 7a). Consistently, cumulative fraction analysis revealed that LUBAC expression significantly increased the abundance of AGO2 targeted mRNAs (RIP covered) (Fig. 7b), indicating that M1-Ubi of AGO2 increased the accumulation of AGO2 bound mRNAs.

In light of the above results, we speculated that the mRNA transcript half-life for decay may be influenced by M1-Ubi of AGO2. Transcripts bound to AGO2 were classified on the basis of published transcriptome-wide half-life measurements^51^, in which the half-life during 0–4 h, 4–12 h and >12 h were categorized as short, middle and long, respectively. Indeed, the abundance of mRNA transcripts with a longer-half-life were significantly much more than those with a shorter-half-life when HOIP and HOIL-1L were expressed (Fig. 7c), suggesting that M1-Ubi of AGO2 critically regulated the half-lives of mRNA transcripts. To further investigate this, HeLa-shHOIP cells were transfected with GFP-4xlet-7a-BS, and then treated with Actinomycin D for indicated times, the expression level of *GFP* mRNA was measured by qRT-PCR to show that knockdown of HOIP greatly shortened the half-lives of *GFP* mRNA (Fig. 7d). Moreover, GO analysis showed that several distinct gene clusters such as translation, DNA repair, cell cycle, mRNA processing and cell division were gathered when HOIP and HOIL-1L were ectopically expressed (Fig. 7e), which coincided with the GO enrichment terms in HeLa cells under hypoxia (Supplementary Fig. 2m). Taken together, our results suggest that M1-Ubi of AGO2 globally inhibits mRNA decay. In addition, we also analyzed the lncRNA expression profiles and found that the increased lncRNA transcripts (186, FPKM > = 30) were 9.3 fold more than the decreased (20, FPKM > = 30) by ectopic expression of HOIP and HOIL-1L (Fig. 7f, Supplementary Data 3), suggesting that M1-Ubi of AGO2 regulates turnover of not only mRNAs but also lncRNAs.

### Hypoxia promotes accumulation of miRNA-targeted mRNAs in clinical tumor specimens

**Fig. 8:**
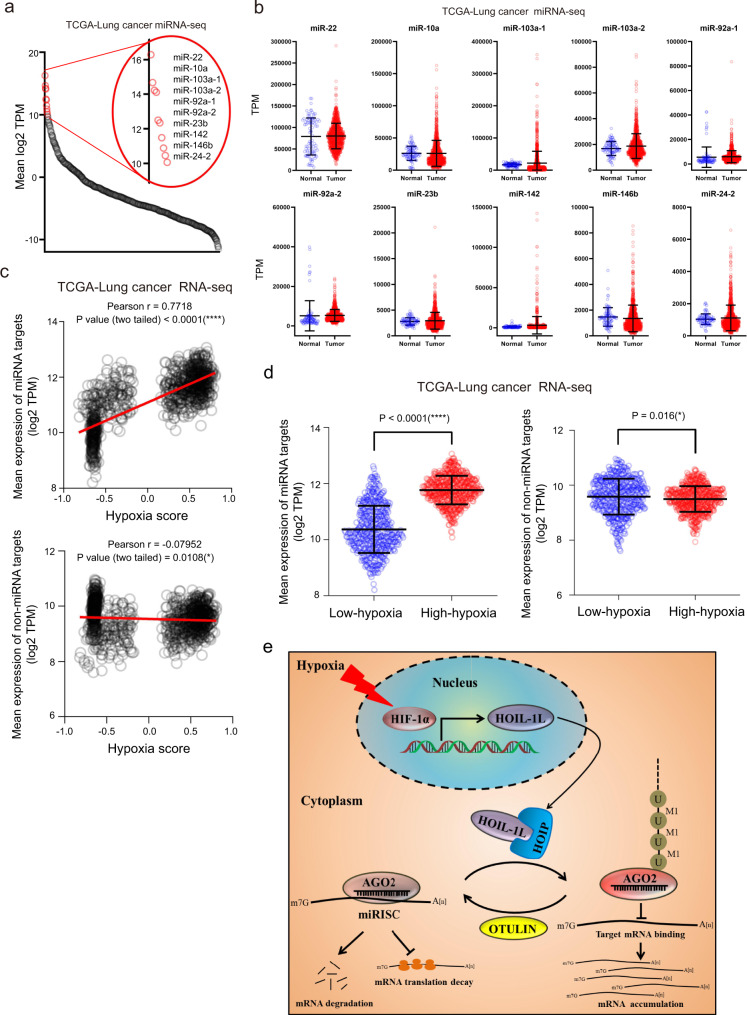
Hypoxia promotes accumulation of miRNA-targeted mRNAs in clinical tumor specimens. **a**, **b** Analysis of all (**a**) and top 10 (**b**) non-difference miRNAs between normal tissues (*n* = 91) and lung cancer tissues (*n* = 999) from TCGA lung cancer miRNA-seq data. **c** The expression levels of the top 10 non-difference miRNA-targeted mRNAs were positively correlated (Pearson *r* = 0.7718, *P* value (two tailed) < 0.0001(****)) and the non-miRNA targets were slightly negative correlated (Pearson *r* = −0.07952, *P* value (two tailed) = 0.0108(*)) with hypoxia scores from TCGA lung cancer (*n* = 1028) RNA-Seq data, respectively. **d** The expression levels of the top 10 non-difference miRNA targets and the non-miRNA targets in high-hypoxia lung cancer tissues were much higher and slightly lower than those in low-hypoxia lung cancer tissues, respectively. Clinical lung cancer samples were divided into two groups, low-hypoxia (hypoxia score < 0, *n* = 457) and high-hypoxia (hypoxia score > 0, *n* = 571), according to the hypoxia score. Data were mean ± s.d., and *P*-values were determined by unpaired two-sided t-test. **e** Mechanistic model summarizing how hypoxia in the tumor microenvironment was an incentive for mRNA accumulation by inducing AGO2 M1-Ubi. Briefly, Hypoxia induces HOIL-1L transcription and enhances LUBAC interacting with AGO2, thereby catalyzing M1-Ubi of AGO2, which can be conversely removed by OUTLIN. M1-Ubi of AGO2 suppresses miRNA-targeted mRNAs loading to AGO2, thus to inhibit the miRISC activity, consequently augment global-mRNA accumulation.

To investigate whether hypoxia-induced M1-Ubi of AGO2 promoting mRNA accumulation is also an ubiquitous phenomenon in clinical tumor specimens, TCGA data of miRNA-Seq and RNA-Seq in lung cancer samples were employed. Firstly, we compared the expression levels of miRNAs in lung cancer tissues (*n* = 999), where are regarded as a relative higher hypoxia degree, with those of normal tissues (*n* = 91), to find that the top 10 non-difference miRNAs including mir-22, mir-10a, mir-103a-1, mir-103a-2, mir-92a-1, mir-92a-2, mir-23b, mir-142, mir-146b and mir-24-2 were almost not changed between normal tissues and lung cancer tissues (Fig. 8a, b). Further, RNA-Seq data in lung cancer samples (*n* = 1028) were analyzed to estimate the hypoxia degree by using the method of GSVA (Gene set enrichment analysis)^52^ with a hypoxia metagene signature^53^. The results showed that hypoxia scores were very positively correlated with the expression levels of the top 10 non-difference miRNA-targeted mRNAs (Pearson *r* = 0.7718, *P* value (two tailed) < 0.0001(****)) (Fig. 8c, upper panel) whereas slightly negative correlated with the expression levels of the non-miRNA targets (Pearson *r* = −0.07952, *P* value (two tailed) = 0.0108(*)) (Fig. 8c, lower panel). Moreover, we divided these lung cancer samples as two groups, low-hypoxia (hypoxia score < 0, *n* = 457) and high-hypoxia (hypoxia score > 0, *n* = 571), according to the hypoxia score. The results showed that the expression levels of the top 10 non-difference miRNA targets in high-hypoxia lung cancer tissues were much higher than those in low-hypoxia lung cancer tissues (Fig. 8d, left panel), but the expression levels of the non-miRNA targets were not much different between high-hypoxia and low-hypoxia lung cancer tissues (right Fig. 8d). Collectively, these results demonstrate that hypoxia scores in clinical tumor specimens are positively correlated with the expression levels of miRNA-targeted mRNAs.

## Discussion

Our results find that hypoxia can lead to increase in the expression levels of thousands and thousands of miRNA-targeted mRNAs in tumor cells; however, none of the currently known molecular mechanisms can well explain it. One classical model is that the transcription factor HIF-1α upregulation induced by hypoxia, but which gives rise to the transcriptions of only more than 100 downstream genes in cells^11^. Accordingly, we have raised a key scientific question: How does hypoxia in tumor cells regulate overall mRNA degradation and homeostasis, and how does this regulation play a key role in tumor phenotype? In this study, we answer those questions and reveal a mechanism that hypoxia in the tumor microenvironment is an incentive for mRNA turnover through inducing M1-Ubi modification of the key protein AGO2 in controlling the efficiency of the miRNA pathway (Fig. 8e). Briefly, hypoxia induces the expression of HIF-1α, which up-regulates the transcription of HOIL-1L, the core component of LUBAC. The increased HOIL-1L with its partner HOIP is recruited to and catalyze AGO2 for M1-Ubi, which promotes AGO2 to form a functional hindered rather than normal miRISC. Subsequently, miRNA-targeted mRNAs cannot be loaded to the M1-Ubi modified AGO2/miRISC, thus the overall miRNA-targeted mRNAs are not degraded and stabilized in hypoxic tumor cells. By this mechanism, short-term hypoxia stress may protect overall mRNAs and enhances stress tolerance, whereas long-term hypoxia stress as like in tumor cells results in serious changing of the entire gene expression profile, which is a driving force in the dynamic process of cell malignant evolution. In addition, we find that M1-Ubi of AGO2 can be de-linear-ubiquitinated by OUTLIN.

Although there are many literatures in M1-Ubi, LUBAC and its related substrates are almost focused on inflammation and immune signaling. Increasing evidence suggest that M1-Ubi is involved in non-inflammatory and non-immunological signaling and other functions. LUBAC is recruited to TDP-43 and SOD1 of Htt-polyQ aggregates thus mediating their M1-Ubi, which plays a great role in regulating intracellular stability and proteotoxicity of these proteins implicated in neurodegenerative disease^41^. M1-Ubi also plays a role in controlling chromosome alignment during mitosis, in which LUBAC catalyzes M1-Ubi of the kinetochore motor CENP-E and then regulates its localization at attached kinetochores for the following chromosome alignment^42^. Furthermore, M1-Ubi of STAT1 can inhibit its binding to the type-I interferon receptor IFNAR2, thereby restricting STAT1 activation and controlling antiviral interferon signaling^40^. Here we reveal the function of M1-Ubi in controlling miRNA-targeted mRNA recruiting to AGO2 and modulating mRNA accumulation under hypoxia. Previous researches have revealed that M1-Ubi at different substrates implement diverse functions by mainly changing the protein-protein interactions^17,38,39^. Interestingly, we demonstrate that M1-Ubi acts as a crucial mode in the miRNA pathway through influencing the RNA-protein interaction (which is mRNA recruitment to AGO2 protein) under hypoxia.

The active miRISC formation includes several steps: first, miRNA is loaded to AGO2; second, miRNA-targeted mRNA is recruited to AGO2/miRNA by 3′-untranslated region complementarily pairing with miRNA; third, TNRC6/GW182 is then bound to the complex of AGO2/miRNA/mRNA in a manner of phase separation and the active miRISC is finally established for mRNA silencing. Several factors participate in this process: (1) some RNA binding proteins (RBP) associate directly with miRISC or bind to the same targeted mRNA adjacent to the miRISC location. For examples, HuR, an AU-rich element-binding protein, interacts with cationic amino acid transporter 1 (CAT-1) mRNA near to the miR-122-RISC location and inhibits its activity under amino acid starvation^54^. FUS promotes the activity of miRNA-mediated gene silencing through direct association with the miRISC components including AGO2, miRNAs and targeted mRNAs, for optimal miRNA-mediated gene silencing^55^. (2) Co-factor proteins directly interact AGO2, such as nucleoporin Nup358 interacts with AGO2 and promotes the association of targeted mRNA with AGO2/miRNA^56^. Eukaryotic translation initiation factor (eIF1A) interacts with AGO2 and augments AGO2-mediated DICER-independent miRNA biogenesis and miRNA-mediated gene silencing^57^. (3) Post-translational modifications of AGO2 also take responsibility for this process. S-Nitrosylation of AGO2 at Cys691 by microbiome-derived NO increases its interaction with TNRC6/GW182 for miRNA-mediating gene expression^58^. Phosporylaiton of AGO2 at Tyr529 impairs the miRNA loading^59^, and this tyrosine residue within the 5′-phosphate-binding pocket of the MID domain has been found directly associated with the first nucleotide phosphate group of miRNA on the molecular crystal level^60^. Akt-mediated phosphorylation of AGO2 at S387 inhibits its association with GW182 and then suppresses miRNA-mediated gene silencing^61^. Phosphorylation of AGO2 at S824-S834, located at a structurally unresolved loop of PIWI domain^60^, impairs its interaction with target mRNA and subsequently suppresses miRNA-mediated gene silencing^62,63^. It is important to find a unrevealed modification of the core molecule AGO2 elucidating the mechanism under specific cellular conditions, such as hypoxia. We identify AGO2 can occur M1-Ubi modification, which is majorly and functionally at K820 that is very close to the phospho-site region S824-834. The M1-Ubi modified AGO2/miRISC is functionally hindered to recruit miRNA-targeted mRNAs, thus blocking the overall mRNA decay.

## Methods

### Cell cultures and transfection

Human embryonic kidney (HEK) 293T (Cat#TCHu150), HeLa (Cat#TCHu187), DU145 (Cat#TCHu222), H1299 (Cat#TCHu160), A549 (Cat#TCHu150), PC3 (Cat#TCHu158) were purchased from the Stem Cell Bank, Chinese Academy of Sciences, Shanghai, China. BPH1 is a gift from Prof. Simon W. Hayward of Vanderbilt University. Cells were cultured in Dulbecco’s modified Eagle’s medium (DMEM, Corning) containing 10% fetal calf serum (FBS, GIBCO), penicillin and streptomycin (Invitrogen) at 37 °C with 5% CO_2_. PC3 cells were cultured in RPMI1640 (Corning) containing 10% FBS, penicillin and streptomycin at 37 °C with 5% CO_2_. All transfections were performed by using Lipofectamine 2000 (Invitrogen).

### Antibodies and reagents

Antibodies against AGO2 (#2897; 1:1000), Myc (#2276; 1:1000), DICER (#3363; 1:1000), PTEN (#9188; 1:1000) and GFP (#2956; 1:2000) were from Cell Signaling Technology. Monoclonal antibody against linear ubiquitin LUB9 clone (#AB130; 1:1000) was from LifeSensors. Monoclonal antibodies against AGO2 (#MABE253; 1:1000), linear ubiquitin 1E3 clone (#MABS199; 1:1000) were from Millipore. Antibodies against AGO2 (#ab57113; 1:250), HOIL-1L (#ab38540; 1:250), OTULIN (#ab151117; 1:250) were from Abcam. Monoclonal antibodies against DGCR8 (#60084-1-Ig; 1:1000), TARBP2 (#15753-1-AP; 1:1000), His-tag (#66005-1-Ig; 1:1000), GAPDH (#60004-1-Ig; 1:3000), Tubulin (#66031-1-Ig; 1:1000) and SHARPIN (#14626-1-AP; 1:1000) were from Protein Tech Group. Antibody against HIF-1a (#NB-100-479; 1:1000) was from Novusbio. Monoclonal antibodies anti-Flag M2 (#F1804; 1:1000) and anti-HA (#A448-101L; 1:1000) were from Covance. Antibodies against normal mouse IgG sc-2025 (1:250), normal rabbit IgG sc-2027 (1:250), GW182 sc-56314 (4B6; 1:500), HOIL-1L sc-393754 (H-1; 1:500) were from Santa Cruz Biotechnology. Antibody against GST (#CW0084; 1:2000) was from CWbioTech (Shanghai, China). ProteinG Plus/ProteinA agarose suspension (#IP05) was purchased from Calbiochem. Glutathione Sepharose 4B (#17-0756-01) was from GE Healthcare Life Sciences (USA). Antibody against HOIP (#SAB2102031; 1:1000), polybrene (hexadimethrine bromide; #H9268) and antibiotic puromycin (#P8833) were from Sigma. Dual-luciferase Reporter Assay System reagent (#E1960) was from Promega. Chemiluminescent Nucleic Acid Detection Module (#89880) was from Thermo Scientific. Let-7a mimics, siPTEN, siHOIP and siOTULIN were obtained from GenePharma (Shanghai, China). Sequences for miRNA mimics, siRNAs and primers were listed in Supplementary Table 1.

### Plasmids

Plasmids pCS2-Myc_6_-AGO2, CD513B-Flag-AGO2 and GST-AGO2 are previously described^64^. AGO2 cDNA was cloned from pCS2-Myc_6_-AGO2 into the vector pEGFP-C1 to get the construct of pEGFP-C1-AGO2. HOIP, SHARPIN, HOIL-1L and OTULIN cDNAs were subcloned to the vectors pCMV-Myc, CD513B or pET-28a, respectively. Point mutations and functional domain deletions for AGO2, HOIP, SHARPIN, HOIL-1L and OTULIN were generated by using the KOD-plus-mutagenesis Kit (TOYOBO). HOIP and HOIL-1L were subcloned to the vector mCherry-N1 to generate constructs mCherry-N1-HOIP and mCherry-N1-HOIL-1L, respectively. shRNA oligoes for HOIP, SHARPIN and OTULIN were subcloned into the lentiviral vector pLKO.1, building stable cell lines with the packaging plasmids pMD2G and pCMV-dR8. The primary miRNAs including pri-let-7a-3 and pri-miR-451a were cloned into the CD513B vector. Primer sequences for plasmid construction and shRNA sequences were listed in Supplementary Table 1.

### Protein purification

The prokaryotic expression constructs pEGX-4T1-AGO2, pEGX-4T1-UBAN, pEGX-4T1-MS2, pET-28a-HOIP, pET-28a-HOIL-1L and pET-28a-SHARPIN were transformed into BL21 competent cells with 0.5 mM isopropyl β-d-1-thiogalactopyranoside (IPTG) inducing for 12–16 h at 16 °C. (1) For GST-AGO2, GST-UBAN and GST-MS2 purifications, bacteria were lysed in B-PER Protein Extraction Reagent (#78248, Thermo Fisher, USA) with 100 μg/ml lysozyme (Sigma), 12.5 μl/ml protease inhibitor cocktails (Sangon) and 2 U/ml DNase I (Thermo Fisher) for 1 h at room temperature. GST-fusion proteins were purified with Glutathione sepharose 4B beads (GE healthcare) and gradually eluted with 20 mM GSH (reduced glutathione; 50 mM Tris pH8.0) and 10 mM GSH for 15 min. (2) For His-tagged HOIP, HOIL-1L and SHARPIN purification, bacteria were harvested and lysed by sonication in the buffer consisting of 50 mM Tris-HCl pH 8.0, 300 mM NaCl. The inclusion bodies were harvested by centrifugation at 13,000 g for 20 min at 4 °C. After washing twice with 20 ml ice-cold wash buffer (2 M urea, 50 mM Tris-HCl pH 8.0 and 300 mM NaCl), the inclusion bodies were dissolved in 10 ml of lysis buffer consisting of 6 M urea, 50 mM Tris-HCl pH 8.0 and 300 mM NaCl. The denatured proteins were refolded after being dialyzed twice in 2 L ice-cold buffer containing 50 mM Tris-HCl pH 8.0 and 150 mM NaCl. Soluble refolded His_6_-HOIP, His_6_-HOIL-1L and His_6_-SHARPIN were further concentrated by using ultra centrifugal filters. (3) For purification of Flag-AGO2, 293T cells were transfected with Flag-AGO2 for 48 h, and then performed with immunoprecipitation in RIPA lysis buffer (50 mM Tris-HCl pH7.4, 150 mM NaCl, 1% NP-40, protease inhibitor cocktail (Roche)) by Flag-M2 beads, the beads bound with Flag-AGO2 were washed by high salt RIPA wash buffer (50 mM Tris-HCl pH7.4, 500 mM NaCl, 1% NP-40, protease inhibitor cocktail) for 3 times. Flag-AGO2 protein was purified by 3xFlag peptide (Sigma-Aldrich) according to the manufactory’s instruction and washed in a non-denaturing high salt buffer (500 mM NaCl).

### In vitro AGO2 M1-Ubi assay

Purified Flag-AGO2 was co-incubated with purified ubiquitin and LUBAC components proteins including HOIP, HOIL-1L and SHARPIN within in vitro M1-Ubiquitin reaction system as follow: 30 μl of reaction buffer (50 mM Tris-HCl, pH 8.0, 150 mM NaCl, 10 mM MgCl_2_, 0.1 mM DTT and 10 mM ATP) containing 3 μg of ubiquitin, 300 ng of UBA1, 800 ng of UBCH5C, 300 ng of Flag-AGO2 and 1 μg of His_6_-HOIP, 1 μg of His_6_-HOIL-1L or/and 1 μg of His_6_-SHARPIN (LUBAC components). The reactions were incubated at 37 °C for 6 h and stopped by the addition of SDS-PAGE protein loading buffer containing DTT and subsequently divided into two samples, one sample was immunoblotted with anti-Flag antibody for the detection of AGO2 M1-Ubi, the other one was immunoblotted with linear ubiquitin antibody 1E3 clone for the examination of the LUBAC complex activity.

### Determination of M1-Ubi of AGO2 under denaturing condition

HeLa cells treated with hypoxia as indicated times were lysed with denaturing lysis buffer (50 mM Tris-HCl pH 7.4, 150 mM NaCl, 2% SDS, 5 mM DTT and Protease inhibitor cocktail) for 10 min, and then lysates were boiled at 95 °C for 10 min and sonicated. Subsequently, cell lysates were centrifuged at 16,000 g for 30 min at 4 °C, then supernatant lysates were diluted 1:20 with lysis buffer (50 mM Tris-HCl pH 7.4, 150 mM NaCl, 5 mM DTT, 1% NP-40 and protease inhibitor cocktail) for the following immunoprecipitation (IP) with anti-AGO2 antibody and incubated with Protein A/G beads overnight at 4 °C. Beads were washed five times with lysis buffer without protease inhibitor cocktail, and then dissolved in SDS-PAGE protein loading buffer containing DTT and denatured at 95 °C for 10 min, a part of the samples were measured by Western blot for AGO2 M1-Ubi analysis. Meanwhile, the remained samples were subjected to SDS-polyacrylamide gel for coomassie brilliant blue staining, the gels containing linear ubiquitinated AGO2 were cut and digested with trypsin for mass spectrometry analysis.

### Co-immunoprecipitation (Co-IP)

293T or HeLa cells transfected with indicated plasmids were lysed with RIPA lysis buffer (50 mM Tris-HCl pH7.4, 150 mM NaCl, 1% NP-40, protease inhibitor cocktail). Cell lysates were incubated with indicated antibodies and protein A/G-agarose beads at 4 °C overnight, and then washed for five times with RIPA lysis buffer without protease inhibitor cocktail and followed by Western blotting analysis.

### M1-SUB pull-down assay

293T cells were transfected with indicated plasmids or treatment of hypoxia, and then cells were lysed with RIPA lysis buffer (50 mM Tris-HCl pH7.4, 150 mM NaCl, 1% NP-40, protease inhibitor cocktail) on ice for 1 h. Lysates were incubated with purified GST-UBAN protein and Glutathione sepharose 4B beads at 4 °C overnight. Linear ubiquitinated AGO2 bound to GST-UBAN-beads were washed for three times by using RIPA lysis buffer, and followed by Western blotting analysis.

### RNA immunoprecipitation (RIP) and qRT-PCR analysis

The methods were modified from our previous study^64^. Briefly, stable cell lines were treated with or without hypoxia, and then were lysed in RIP-lysis buffer (50 mM Tris-HCl pH7.4, 150 mM NaCl, 10 mM EDTA, 5 mM MgCl_2_, 1% NP-40, 1 mM DTT, 100 units/ml RNase inhibitor (Fermentas), 400 μM VRC (New England BioLabs) and Protease inhibitor cocktail) on ice for 1 h. 1/10 of lysates were extracted with 1 ml of TRIZOL reagent (Invitrogen) for extraction of total RNAs as input, 1/50 of lysates were saved for Western blotting to detect the protein expression, and left lysates were incubated with indicated antibodies and protein A/G-agarose beads at 4 °C overnight. Beads bound with RNAs were washed with the same RIP-lysis buffer for five times, then 1/10 of beads was subjected to Western blotting analysis to identify the efficiency of IP, the remained beads were extracted with 1 ml of TRIZOL reagent for extraction of RIP bound RNAs.

For RIP-qRT-PCR, RNAs extracted from RIP bound or total input RNAs were firstly treated with DNase I (Thermo) to degrade genomic DNA. Reverse transcription was performed by using PrimeScript^TM^ RT-PCR Kit (TAKARA), oligo-dT primers and random primers were used to reverse transcript. SYBR^®^ Green PCR Master Mix (Applied Biosystems) was used for qPCR on StepOne Plus Real-Time PCR System (Applied Biosystems) to analyze the RNAs bound to AGO2; RIP fractions of mRNA were normalized and calculated as % input.

### Let-7a luciferase reporter for miRISC activity assay

Four-repeated let-7a binding site (BS) sequences of 4xlet-7a-BS were inserted into the 3′-UTR of *Renilla* luciferase of the vector psiCHECK2 to constitute psiCHECK2-4xlet-7a-BS. Accordingly, *Renilla* luciferase is expectedly attenuated when let-7a binds to binding sites. AGO2, psiCHECK2-4xlet7a-BS and indicated plasmids or siRNAs were co-transfected into 293T cells, 48 h after transfection the cells were subjected to the dual-luciferase reporter assay according to the manufacturer’s instruction. The miRSIC activity with *Renilla* luciferase was normalized by *Firefly* luciferase.

### GFP expression for miRSIC activity assay

Four-repeated let-7a binding site sequences of 4xlet-7a-BS was inserted into the 3′UTR of *GFP* in the vector of pEGFP-C1 to get pEGFP-C1-4xlet-7a-BS, and then the reporter pEGFP-4xlet-7a-BS was subcloned into the lentiviral vector pCD510 to form pCD510-GFP-4xlet-7a-BS for construction of stable cell lines. HeLa or 293T cells were transfected with pEGFP-C1-4xlet-7a-BS and indicated plasmids or siRNAs, 48 h after transfection cells were collected for Western blotting and qRT-PCR analyses of GFP in protein level and mRNA level, respectively.

### HOIL-1L promoter luciferase activity assay

The HOIL-1L promoter region (2.0 kb upstream of transcription start site) was cloned into the vector pGL3-Basic-luciferase reporter (Promega) to get pGL3-HOIL-1L-P. 293T cells were seeded on 24-well plates and co-transfected with the *Renilla* luciferase reporter pSV-40 and pGL3-HOIL-1L-P or pGL3-Basic. 48 h later the luciferase activity was measured by dual-luciferase reporter assay according to the instruction, the HOIL-1L promoter luciferase was normalized by *Renilla* luciferase activity.

### Mass spectrometry (MS) analysis

For identification of the proteome profile associated by AGO2 under hypoxia, HeLa cells stably expressing Flag-AGO2 were treated with normoxia (21% O_2_) and hypoxia (1% O_2_) for 24 h, respectively. For identification of potential M1-Ubi sites of AGO2, 293T cells were co-transfected with HA-AGO2 and Flag-HOIP/HOIL-1L for 48 h. Cells were lysed with RIPA lysis buffer (50 mM Tris-HCl pH7.4, 150 mM NaCl, 10 mM EDTA, 1% NP-40, protease inhibitor cocktail tablet), then lysates were incubated with anti-Flag M2 monoclonal antibody or anti-HA and protein A/G-agarose beads at 4 °C overnight. Beads were washed for five times with the same RIPA lysis buffer, and then dissolved in 10% SDS lysis buffer, denatured at 95 °C for 10 min. For the proteome profile, the samples were directly used for trypsin digestion step; for the M1-Ubi sites, the samples were subjected to SDS-polyacrylamide gel for coomassie brilliant blue staining, the gel containing linear ubiquitinated AGO2 was cut for trypsin digestion step.

For mass spectrometry analysis, Nano-LC-MS/MS was used^65^. Protein samples were gradually reduced and alkylated by dithiothreitol (DTT) in a final concentration of 10 mM at room temperature for 1 h and iodoacetamide in a final concentration of 55 mM at room temperature for 1 h in the dark conditions, respectively. Next, the samples were digested by sequencing-grade modified trypsin (Promega) at 37 °C overnight, and then collected by centrifugation for 20 min at 14,000 *g* at room temperature. The tryptic peptides were treated with 1% trifluoroacetic acid, purified by using C18 Ziptips and eluted with 0.1% trifluoroacetic acid in 60% acetonitrile. The eluted peptides were lyophilized using a SpeedVac (ThermoSavant), and resuspended in 10 μl of 1% formic acid/5% acetonitrile. All mass spectrometric experiments were performed on an Orbitrap Fusion LUMOS mass spectrometer (Thermo Fisher Scientific) connected to an Easy-nLC 1200 via an Easy Spray (Thermo Fisher Scientific). The reverse-phase microcapillary column (0.1 × 150 mm) packed with Reversed Phase C18 resins (2 μm, PepMap RSLC) was used for mixed peptides loading at a flow of 1 μl/min. Then a 60-min linear gradient solution from 95% buffer A (0.1% formic acid, 2% acetonitrile and 98% water) to 30% buffer B (0.1% formic acid and 80% acetonitrile) at a flow rate of 0.3 μl/min was conducted for peptides reverse-phase separation. The radio frequency (RF) lens was 60%, mass spectrometer was operated in positive ion mode and employed in the data-dependent mode within the specialized cycle time (3 s), to automatically switch between mass spectrometry and tandem mass spectrometry (MS/MS). One full mass spectrometry scan from 350-1, 500 m/z was acquired at high resolution (*R* = 120,000 (defined at *m/z* = 400)). MS/MS scans were acquired at a resolution of 30,000. Masses selected for MS/MS were isolated (quadrupole) at a width of 4 Da and were fragmented using a higher energy collisional dissociation of 30% ± 5. All MS/MS ion spectra were analyzed by using PEAKS 10.0 (Bioinformatics Solutions) for processing, de novo sequencing and database searching. Resulting sequences were searched against the UniProtHuman Proteome database. Carbamidomethyl (57.02 Da) of cysteine was specified as a fixed modification, oxidation (15.99 Da) of methionine, acetylation (42.01 Da) of the N terminus and ubiquitination (114.04 Da) of lysine were specified as variable modifications. FDR estimation was enabled. Peptides and proteins were separately filtered for −log10(*P* value) ≥ 20 and for −log10(*P* value) ≥ 15. For all of the experiments, this gave an FDR < 1% at the peptide-spectrum match level. Proteins sharing significant peptide evidence were grouped into clusters. The relative ratios (hypoxia/normoxia) of proteins associated with AGO2 under hypoxia and normoxia conditions were calculated by normalizing to AGO2 itself. The mass spectrometry (MS)-based proteomics was performed at the Proteomics of Core Facility of Basic Medical Sciences, Shanghai Jiao Tong University School of Medicine (SJTU-SM).

### Immunofluorescence staining

For immunofluorescence (IF) staining^66^, HeLa cells, stable HeLa cells expressing of HOIP/HOIL-1L and HeLa cells knocking down of HOIP or OTULIN were cultured on glass coverslips in 24-well plates, and then transfected with indicated plasmids. 24 h after transfection the cells on coverslips were washed twice times with PBS and immobilized by 4% PFA for 30 min at room temperature. After washing three times with PBS (5 min each time), cells were blocked by blocking solution (5% BSA in TBS with 0.5% Triton X-100) for 1 h for the following incubation with antibodies against TNCR6A/GW182, HOIP, HOIL-1L and HA in blocking solution for 1 h at room temperature. Moreover, cells were washed three times with PBS (5 min each time) and then incubated with appropriate secondary antibodies conjugated with AlexaFluorescence 568 or with AlexaFluorescence 647 in blocking solution for 1 h at room temperature away from light. Furthermore, after washed three times with PBS (5 min each time), the cells were stained with DAPI for 30 min in the dark. Finally, IF images were captured using a laser scanning confocal microscopy.

### High-throughput sequencing for RIP-Seq, RNA-Seq and miRNA-Seq

For RIP-Seq, indicated stable HeLa cell lines were conducted by RNA immunoprecipitation (RIP) for the following sequencing were performed as described^67^. RNAs bound to AGO2 and total RNAs (as an input) were extracted by using TRIZOL reagent as following manufacturer’s instruction (Invitrogen), then the rRNAs were removed from the immunoprecipitated RNA and input RNA samples by using RNAs with NEBNext rRNA Depletion Kit (New England Biolabs, Inc., Massachusetts, USA). The rRNA-depleted RNAs were constructed RNA sequencing libraries by using NEBNext^®^ Ultra™ II Directional RNA Library Prep Kit (New England Biolabs, Inc., Massachusetts, USA) according to the manufacturer’s instructions.

For RNA-Seq, total RNA (1 μg) extracted from indicated stable HeLa cell lines by TRIZOL reagent was used for removing the rRNAs using Ribo-Zero rRNA Removal Kits (Illumina, San Diego, CA, USA) as the manufacturer’s instructions for the following library construction. The rRNA-depleted RNAs were constructed RNA sequencing libraries by using TruSeq Stranded Total RNA Library Prep Kit (Illumina, San Diego, CA, USA) according to the manufacturer’s instructions.

For miRNA-Seq, total RNAs were extracted from indicated stable HeLa cell lines by using TRIZOL reagent. Each extracted RNA was used for the preparation of the miRNA sequencing library, which including 3′-adaptor ligation, 5′-adaptor ligation, cDNA synthesis and PCR amplification, approximately 150 bp PCR amplicons (corresponding to ~22 nt miRNAs) size of products were selected. Constructed RIP-Seq and RNA-Seq libraries were controlled for quality and quantified using the BioAnalyzer 2100 system (Agilent Technologies, Inc., USA), and the libraries sequencing were performed on an illumina Hiseq instrument with 150 bp paired-end reads. Constructed miRNA-Seq libraries sequencing were denatured as single-stranded DNA molecules, captured on Illumina flow cells, amplified in situ as clusters and finally sequenced for 50 cycles on Illumina HiSeq Sequencer according to the manufacturer’s instructions.

High-throughput sequencing for RIP-Seq, RNA-Seq and miRNA-Seq were all done by Cloud-Seq Biotech (Shanghai, China).

### Analyses for High-throughput sequencing data

Paired-end reads were harvested from Illumina HiSeq 4000 sequencer, and were quality controlled by Q30. After 3′ adaptor-trimming and removing of low quality of reads by cut adapt software (version 1.9.3). For RIP-Seq, high quality clean of trimmed reads were aligned to the human reference genome (UCSC hg19) by bowtie2 software (version 2.2.4) with default parameters. The target binding regions of AGO2 were identified using MACS2 software (version 1.4.3). High-confidence binding regions of AGO2 were identified by stringent cutoff threshold, and then annotated with the latest UCSC RefSeq database to connect the peak information with the gene annotation. Fold enrichment of each mRNA transcript matched to different MACS identified motifs were summed. For RNA-Seq, high-quality clean reads were aligned to the reference genome (UCSC hg19) with hisat2 software (version 2.0.4). Then, guided by the Ensembl gtf gene annotation file, cuffdiff software (part of cufflinks) was used to get the gene-level FPKM (Fragments per kilobase of exon per million fragments mapped) as the expression profiles of mRNA, and fold change and *p*-value were calculated based on FPKM, differentially expressed mRNA were identified.

For miRNA-Seq, raw data were generated after sequencing, image analysis, base calling and quality filtering on Illumina sequencer and finally quality controlled by Q30. The adaptor sequences were trimmed and the adaptor-trimmed-reads (>= 15 nt) were left by cut adapt software (version 1.9.2). Then the trimmed reads from all samples were pooled, and miRDeep2 software (version 2.0.0.5) was used to predict novel miRNAs. The trimmed reads were aligned to the merged human pre-miRNA databases (known pre-miRNA from miRBase plus the newly predicted pre-miRNAs) using Novoalign software (version 3.02.12) with at most one mismatch. The numbers of mature miRNA mapped tags were defined as the raw expression levels of that miRNA. The read counts were normalized by TPM (tag counts per million aligned miRNAs) approach. Differentially expressed miRNAs between two samples were filtered through Fold change. miRNA targets were performed by popular miRNA target prediction by using TargetMiner.

### Preparation for Biotin labeled pre-let-7a-3 and HMGA2-3′-UTR-mut

For preparation of pre-let-7a-3^68^, a short RNA contains hammerhead ribozyme linked pre-let-7a-3 was transcribed through T7 RNA polymerase by using an chimeric DNA T7-RNA-polymerase-promoter-hammerhead-ribozyme-pre-let-7a-3 (synthesized from Sangon Biotech, Shanghai, China) as the transcription template, and it was internal tagged with biotin by using Bio-16-UTP through transcription. Then pre-let-7a-3 was auto-cleavaged by hammerhead ribozyme, and purified from urea/PAGE gel.

For HMGA2-3′-UTR-mut, a short DNA containing T7 RNA polymerase promoter and HMGA2-3′-UTR-mut (T7-RNA-polymerase-promoter-HMGA2-3′-UTR-mut) were amplified by PCR with a specific primer from psiCHECK2-HMGA2-3′-UTR as the transcription template. The HMGA2-3′-UTR-mut RNA was internally tagged with biotin by using Bio-16-UTP through T7 RNA polymerase transcription, and then purified from urea/PAGE gel.

The purified with 5′-OH and 3′-OH end of pre-let-7a-3 and HMGA2-3′-UTR-mut were dephosphorylated by calf intestine phosphatase (CIP), and then were 5′-end-phosphorylated by using T4 polynucleotide kinase (T4 PNK) and ATP. Prior to the use of pre-let-7a-3, 5′-end-phosphorylated pre-let-7a-3 was denatured by incubation at 95 °C for 1 min and then was renatured by incubation at 25 °C for 15 min in working buffer (30 mM Tris-HCl pH6.8, 50 mM NaCl, 2 mM MgCl_2_ and 10% glycerol).

### In vitro target mRNA pull-down assay

293T cells transfected with indicated plasmids or treated with or without hypoxia were lysed with RIP-lysis buffer (50 mM Tris-HClpH7.4, 150 mM NaCl, 10 mM EDTA, 5 mM MgCl_2_, 1% NP-40, 1 mM DTT, 100 units/ml RNase inhibitor, 400 μM VRC and Protease inhibitor cocktail). Lysates were incubated with biotin-HMGA2-3′-UTR-mut purified RNA and Dynabeads™ MyOne™ Streptavidin C1 (Invitrogen) at 4 °C overnight. Then the beads centrifuged, the supernatants sample were saved, while the biotin-HMGA2-3′-UTR-mut-bound-beads were washed for five times with RIP-lysis buffer. AGO2 associated with biotin-HMGA2-3′-UTR-mut on beads and saved supernatants were determined by Western blotting analysis.

### In vitro target mRNA binding assay

293T cells transfected with indicated plasmids were lysed with RIP-lysis buffer (50 mM Tris-HCl pH7.4, 150 mM NaCl, 10 mM EDTA, 5 mM MgCl_2_, 1% NP-40, 1 mM DTT, 100 units/ml RNase inhibitor, 400 μM VRC and Protease inhibitor cocktail). Lysates were performed by IP with anti-HA (AGO2) antibody, and then washed for three times with RIP-lysis buffer. Then beads were gradually incubated with let-7a mimics and biotin-HMGA2-3′-UTR-mut for 30 min, and subsequently washed for three times with RIP-lysis buffer. Biotin-HMGA2-3′-UTR-mut associated with AGO2 on beads were extracted by TRIZOL reagent and examined by Northern blotting analysis.

### In vitro pre-let-7a-3 processing

293T cells transfected with indicated plasmids were lysed with RIP-lysis buffer (50 mM Tris-HCl pH7.4, 150 mM NaCl, 10 mM EDTA, 5 mM MgCl_2_, 1% NP-40, 1 mM DTT, 100 units/ml RNase inhibitor, 400 μM VRC and Protease inhibitor cocktail). Lysates were performed by IP with anti-HA (AGO2) antibody, and then washed for five times with the same RIP-lysis buffer. Beads bound with HA-AGO2 and endogenous DICER were co-incubated within in vitro processing buffer (20 mM Tris-HCl pH 7.5, 3 mM MgCl_2_, 75 mM NaCl, 10% glycerol and RNase inhibitor) at 37 °C for 1 h, pre-let-7a-3 and processed mature let-7a were purified by TRIZOL reagent and following detected by Northern blotting analysis.

### GST-MS2 pull-down assay

Four-repeated let-7a binding site sequences of 4xlet-7a-BS and twelve-repeated MS2 binding site sequences of 12xMS2-BS were subcloned into the vector pcDNA3.1 to form pcDNA3.1-4xlet-7a-BS-12xMS2-BS. The indicated plasmids and pcDNA3.1-4xlet-7a-BS-12xMS2-BS were co-transfected into 293T Cells, then the cells were lysed with RIP-lysis buffer (50 mM Tris-HCl pH7.4, 150 mM NaCl, 10 mM EDTA, 5 mM MgCl_2_, 1% NP-40, 1 mM DTT, 100 units/ml RNase inhibitor, 400 μM VRC and Protease inhibitor cocktail) on ice for 1 h, lysates were incubated with purified GST-MS2 and Glutathione sepharose 4B beads at 4 °C overnight. AGO2 associated with 4xlet-7a-BS-12xMS2-BS bound to beads were washed for three times by using the same RIP-lysis buffer, and followed by Western blotting analysis.

### In vitro RISC assembly assay

293T cells transfected with indicated plasmids were lysed with RISC-lysis buffer (30 mM HEPES-KOH pH7.4, 100 mM KOAc, 2 mM Mg(OAc)_2_, 0.5% NP40), then cell lysates were incubated with let-7a mimics and biotin-HMGA2-3′-UTR-mut in RISC reaction buffer (50 mM creatine monophosphate, 100 μM amino acid solutions, 0.5U/μl RNasin, 5 mM ATP, 1 mM GTP, 100 mM KOAc, 0.1U/μl creatine phosphokinase) at room temperature for 30 min, Biotin-HMGA2-3′-UTR-mut assembled into RISC complexes were performed with native gel and measured by Northern blotting analysis.

### Statistics and reproducibility

Statistical calculations were performed with GraphPad Prism 8 and SPSS analysis tools. *P* values were calculated using a two-sided Mann–Whitney U test for cumulative fraction analysis, qRT-PCR and dual-luciferase reporter assays were presented as means ± s.d. or s.e.m. (*P < 0.05; **P < 0.01; ***P < 0.001; unpaired, two-tailed Student’s *t* test). The mean values obtained in the control and experimental groups were analyzed for significant differences. Unless stated otherwise, the experiments were not randomized and investigators were not blinded to allocation during experiments and outcome assessment. Each of the experiments including Fig. 1c; 2b–l; 3a–l; 4a–d; 5g, h; 6l, n and Supplementary Figs. 1g, h; 2c, j, k; 3a; 4b–n; 5a, e, f; 6a, b, e, f; 7a–g; 8k, l in this study were independently repeated at least three times.

### Reporting summary

Further information on research design is available in the Nature Research Reporting Summary linked to this article.

## Supplementary information

Supplementary Information

Supplementary Data 1

Supplementary Data 2

Supplementary Data 3

Supplementary Data 4

Supplementary Data 5

Supplementary Data 6

Supplementary Data 7

Description of Additional Supplementary Files

Reporting Summary

## Data Availability

The RIP-Seq, miRNA-Seq and RNA-Seq data generated in this study were provided as EXCEL profiles in Supplementary Data and have been deposited in the Gene Expression Omnibus (GEO) repository under the accession number GSE158601. The mass spectrometry data generated in this study were provided as EXCEL profiles in Supplementary Data and have been uploaded to Integrated Proteome Resources (iProX) database under the accession number IPX0002506000. All data are also available from the corresponding author (J.Y.) upon reasonable request. Source data are provided with this paper.

## Source data

Source data
