# Supplementary material for: Hypoxia regulates overall mRNA homeostasis by inducing Met1-linked linear ubiquitination of AGO2 in cancer cells
Source: Nat Commun. 2021 Sep 13;12:5416. doi: 10.1038/s41467-021-25739-5 (PMC8438024; doi:10.1038/s41467-021-25739-5)
Supplement: Supplementary file 1 — Supplementary Information [file 41467_2021_25739_MOESM1_ESM.pdf]

SUPPLEMENTARY INFORMATION

Hypoxia regulates overall mRNA homeostasis by inducing Met[1]-linked linear ubiquitination of AGO2 in cancer cells

Zhnag *et al.*

Supplementary Fig. 1

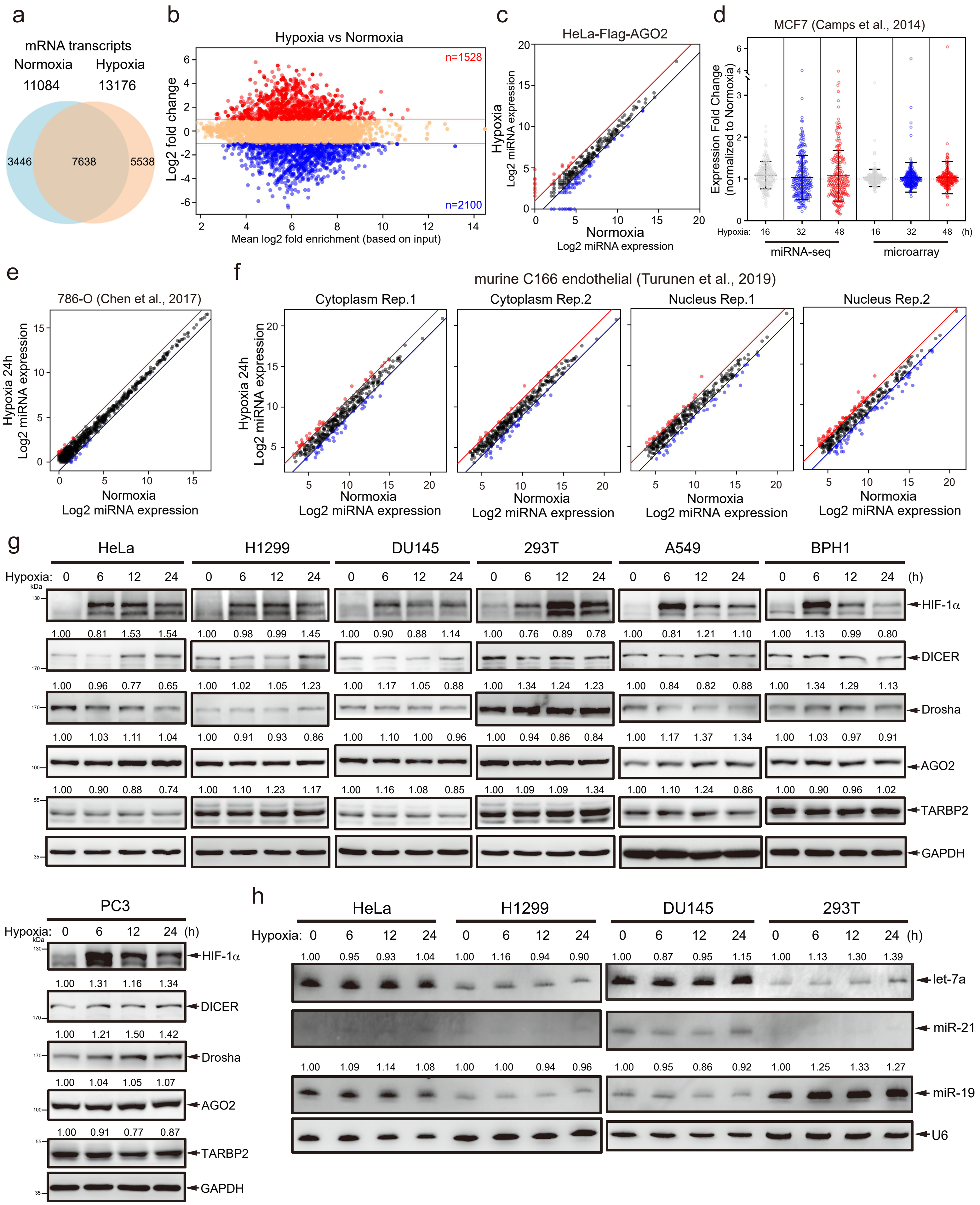

Supplementary Fig. 1 Hypoxia regulates the biogenesis of a fraction of miRNAs. **a** Overlapping of AGO2-associtaed mRNA transcripts identified by RIP-Seq in stable HeLa cells expressing Flag-AGO2 (HeLa-Flag-AGO2) under normoxia (21% O_2) and hypoxia (1% O_2) for 24 h. **b** Scatter distribution plot of mRNA transcripts bound to AGO2 by RIP-Seq (Hypoxia vs Normoxia) in HeLa-Flag-AGO2 cells (Supplementary Data 1). **c** Scatter plot of miRNA expression profiles by high-throughput miRNA sequencing (miRNA-Seq) in HeLa-Flag-AGO2 cells under normoxia (21% O_2) and hypoxia (1% O_2) for 24 h (Supplementary Data 2). **d-f** MiRNA profiles were shown under hypoxia stress. The miRNA expression profiles by miRNA-Seq and microarray of in MCF7 cells (Data were means ± s.d., n=228 miRNAs) (**d**), by miRNA-Seq in 786-O cells (**e**), and by miRNA-Seq of cytoplasm and nucleus in murine C166 endothelial cells (**f**) under hypoxia. **g**, **h** The expression levels of key enzymes in the miRNA pathway and of some miRNAs were slightly affected by hypoxia. HeLa, H1299, DU145, 293T, A549, BPH1 and PC3 cells were individually treated with hypoxia for indicated times, the expression levels of core enzymes in the miRNA pathway were analyzed by WB (**g**) and the expression levels of let-7a, miR-21 and miR-19 were determined by Northern blotting analysis (**h**).

Supplementary Fig. 2

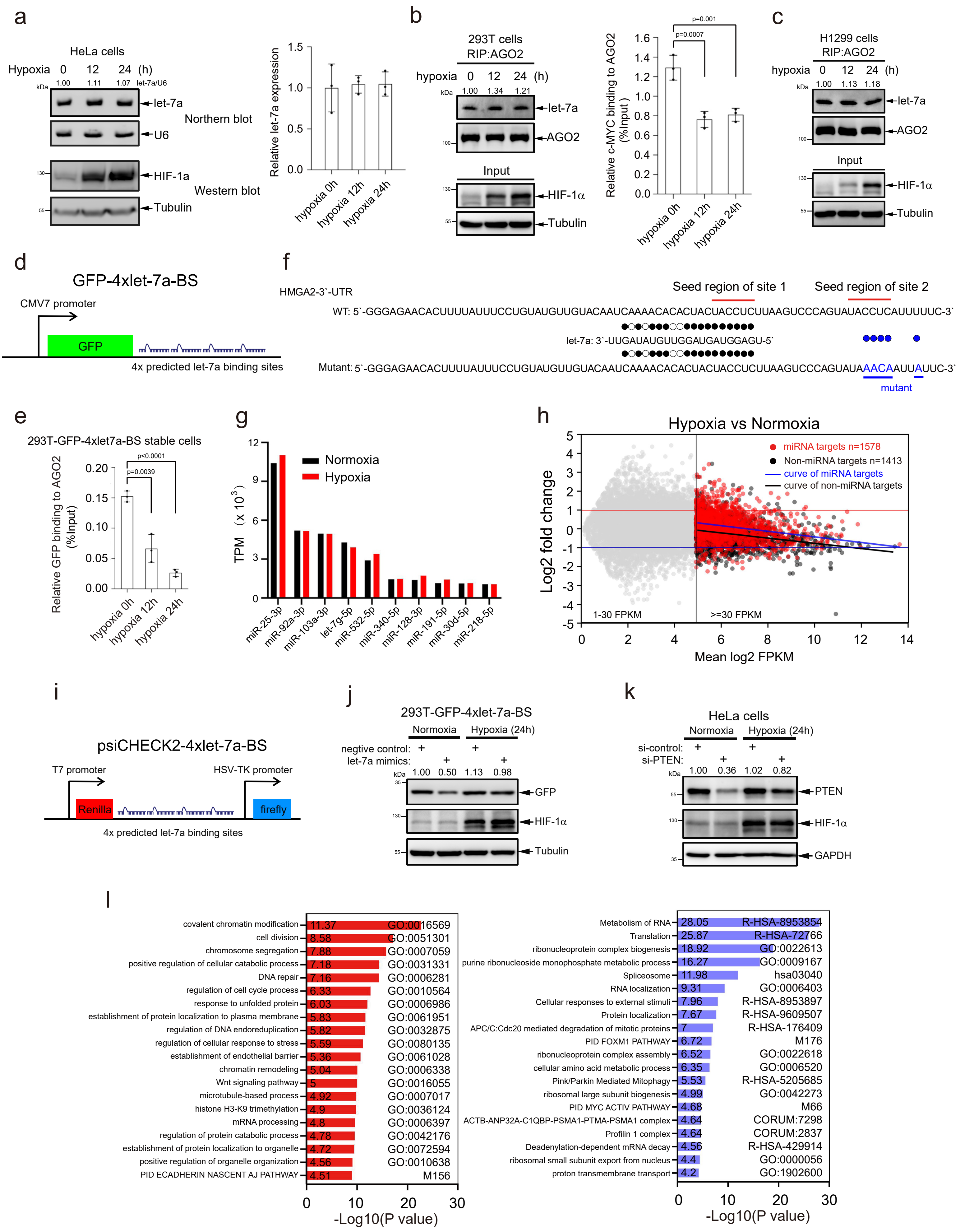

Supplementary Fig. 2 Hypoxia impairs miRNA-targeted mRNA loading to AGO2. **a** The expression level of let-7a was not changed under hypoxia condition. The expression of let-7a was detected by Northern blot and qRT-PCR in HeLa cells. Data were mean ± s.d., n=3 biologically independent samples, p-values were determined by unpaired two-sided t-test. **b-e** Hypoxia attenuated the loadings of let-7a-targeted mRNA but not let-7a to AGO2. Cells were treated with hypoxia for indicated times, and then determined by RNA immunoprecipitation (RIP) assays. The let-7a loading to AGO2 was determined by Northern blotting analysis (**b**, **c**), and the let-7a-targeted mRNAs of *c-MYC* and *GFP* binding to AGO2 were examined by qRT-PCR. Data were mean ± s.e.m., n=3 biologically independent experiments, and *P*-values were determined by unpaired two-sided t-test (**b**, **e**). Diagrams of the let-7a miRISC activity system with constructed GFP expression (**d**). **f** HMGA2-3`-UTR fragment contains two native let-7a target regions, which are over-lined in red. Mutated nucleotides in the seed site 2 are underlined in blue. **g** The expression levels of top 10 non-difference miRNAs profiled by small RNA-Seq in HeLa-Flag-AGO2 under normoxia (21% O_2) and hypoxia (1% O_2). TPM values of each miRNA species and their relative occupancies are shown. **h** Scatter distribution plot of mRNA expression profiles of top 10 non-difference miRNA targets (n=1578) and no miRNA targets (n=1413) by RNA-seq in HeLa-Flag-AGO2 stable cell lines under normoxia and hypoxia. **i** Diagrams of the let-7a miRISC activity with luciferase reporter system. **j** Hypoxia attenuated the let-7a-miRISC activity. 293T cells transfected with GFP-4xlet-7a-BS and let-7a mimics were treated with hypoxia for 24 h, the expression level of GFP was determined by WB. **k** Hypoxia suppressed *PTEN* decay mediated by siRNA. HeLa cells transfected with siRNA targeted to *PTEN* or negative control were treated by hypoxia for 24 h. The expression level of PTEN was determined by WB. **l** Enriched Gene Ontology (GO) categories for genes with 2-fold up regulated (red, left panel) and down regulated (blue, right panel) of mRNA transcripts under hypoxia (Supplementary Data 3).

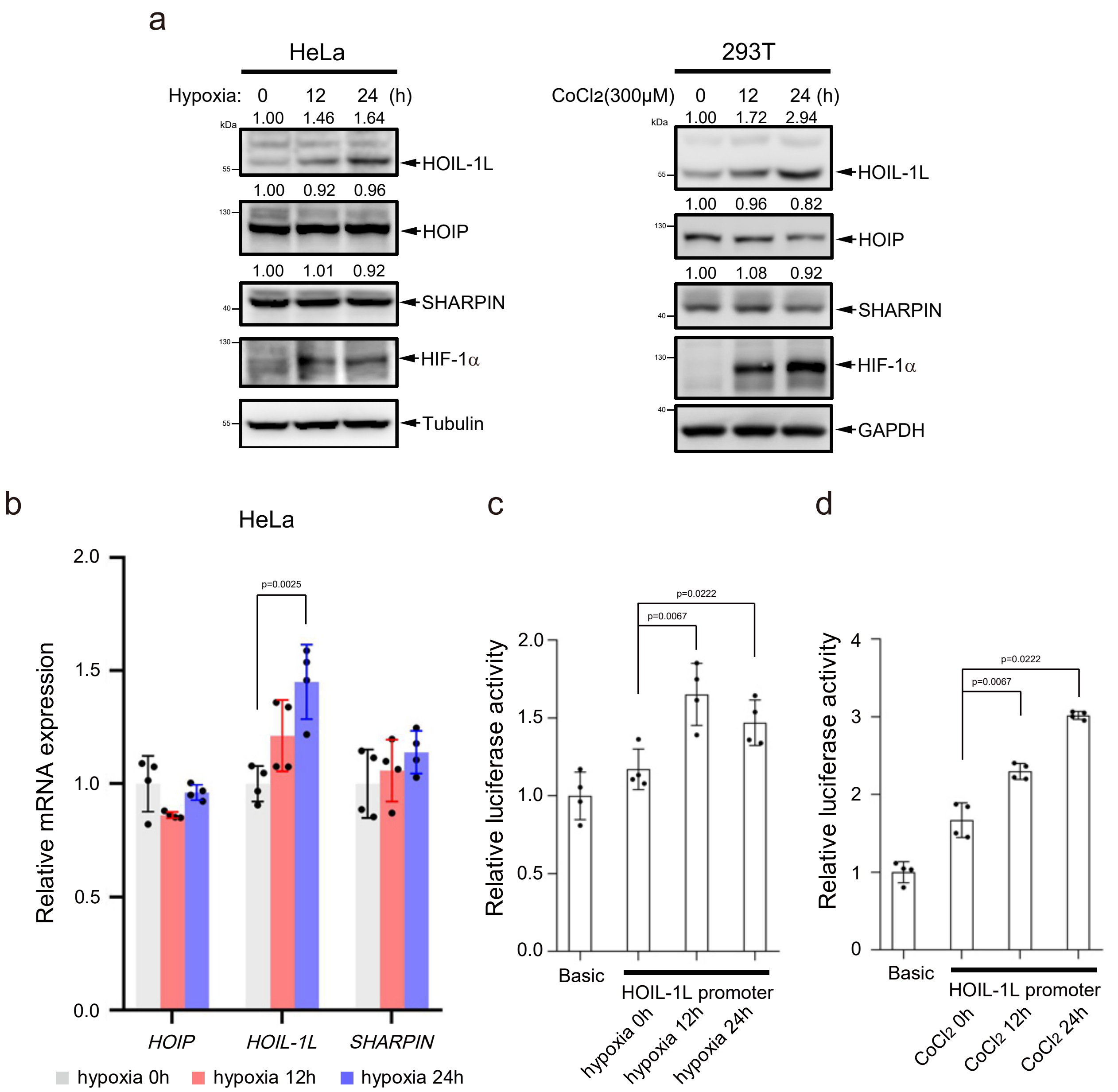
a
HeLa
Hypoxia: 0 12 24 (h)
1.00 1.46 1.64
HOIL-1L
1.00 0.92 0.96
HOIP
1.00 1.01 0.92
SHARPIN
HIF-1α
Tubulin
293T
CoCl2(300μM) 0 12 24 (h)
1.00 1.72 2.94
HOIL-1L
1.00 0.96 0.82
HOIP
1.00 1.08 0.92
SHARPIN
HIF-1α
GAPDH
b
HeLa
Relative mRNA expression
p=0.0025
HOIP
HOIL-1L
SHARPIN
hypoxia 0h
hypoxia 12h
hypoxia 24h
c
Relative luciferase activity
p=0.0222
p=0.0067
Basic
HOIL-1L promoter
hypoxia 0h
hypoxia 12h
hypoxia 24h
d
Relative luciferase activity
p=0.0222
p=0.0067
Basic
HOIL-1L promoter
CoCl2 0h
CoCl2 12h
CoCl2 24h

Supplementary Fig. 3 Hypoxia induces the expression of HOIL-1L. **a** Hypoxia induced HOIL-1L protein expression. HeLa cells were treated with hypoxia (Left panel) and 293T cells were added with 300 μM $CoCl_2$ (Right panel) for the indicated times. The expression levels of HOIL-1L, HOIP, SHARPIN and HIF-1α were detected by WB. **b** Hypoxia induced *HOIL-1L* mRNA transcription. HeLa cells were treated with hypoxia for the indicated times, the mRNA transcript levels of *HOIL-1L*, *HOIP* and *SHARPIN* were determined by qRT-PCR and normalized with *β-actin* mRNA. Data were mean ± s.d., n=3 biologically independent samples, and *P*-values were determined by unpaired two-sided t-test. **c**, **d** Hypoxia increased the luciferase activity of *HOIL-1L* promoter. The *Renilla* luciferase reporter pSV-40 was co-transfected with pGL3-basic or pGL3-HOIL-1L promoter into 293T cells, and then cells were treated with hypoxia (**c**) or $CoCl_2$ (**d**, 300 μM) for indicated times, the luciferase activity of pGL3-HOIL-1L promoter was measured by the dual luciferase assay and normalized with *Renilla* luciferase. Dual luciferase reporter assay data were mean ± s.e.m., n=4 biologically independent samples, and *P*-values were determined by unpaired two-sided t-test.

Supplementary Fig. 4

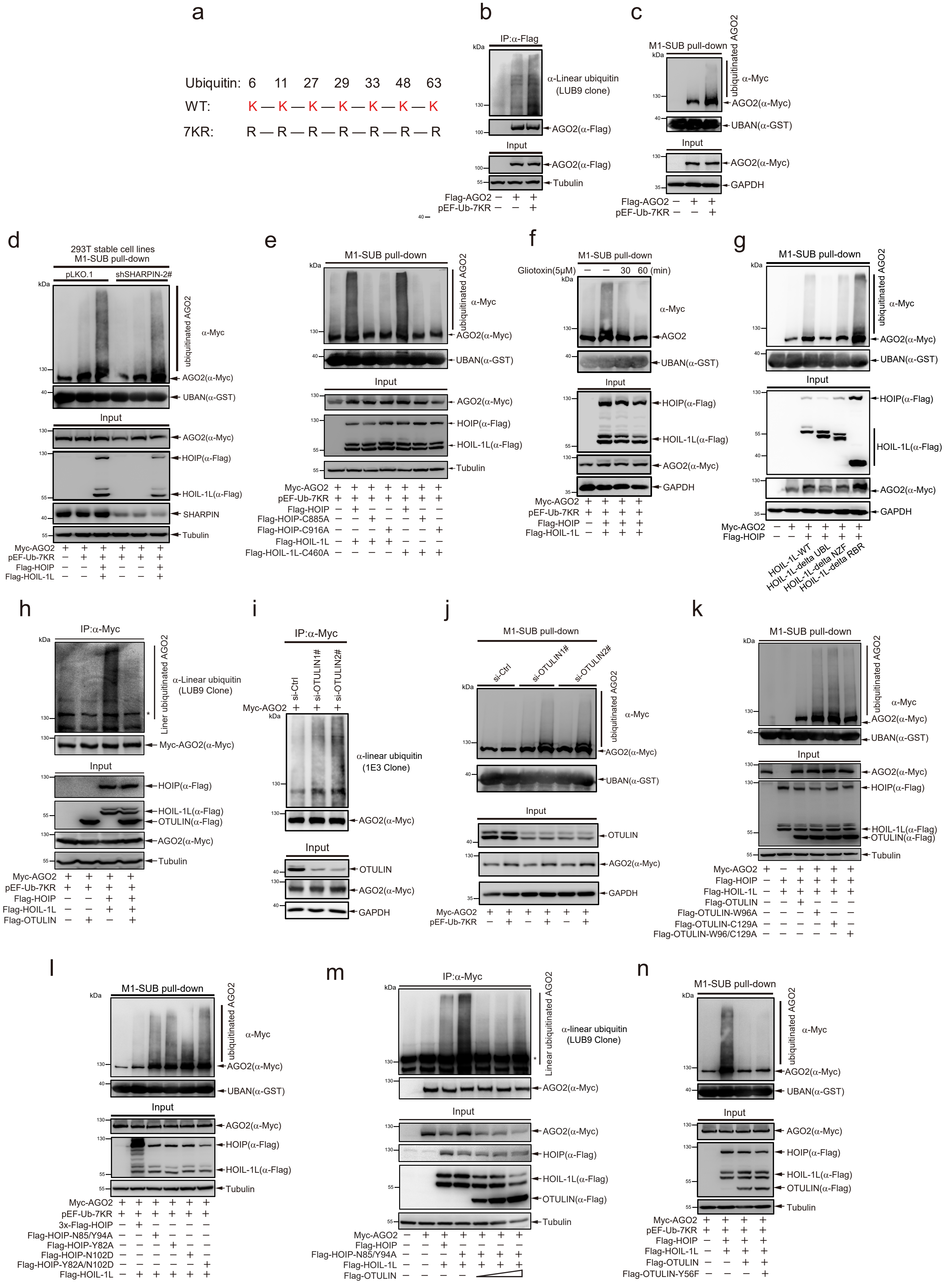

Supplementary Fig. 4 AGO2 M1-Ubi is catalyzed by LUBAC whereas is removed by OUTLIN. **a** Schematic diagram of ubiquitin WT and the mutant 7KR, in which all seven lysines are mutated to arginines. **b**, **c** AGO2 occurred M1-Ubi. 293T cells were co-transfected with Flag-AGO2 and pEF-Ub-7KR, and M1-Ubi of AGO2 was detected by IP/WB with antibody LUB9 clone (**b**) or determined by M1-SUB pull-down/WB (**c**). **d** Knockdown of SHARPIN had little effect on AGO2 M1-Ubi. Stable 293T-shSHARPIN cells were co-transfected with indicated plasmids. AGO2 M1-Ubi was determined by M1-SUB pull-down/WB. **e** AGO2 M1-Ubi was dependent on the catalytic activity of HOIP, but not that of HOIL-1L. 293T cells were co-transfected with indicated plasmids, and M1-Ubi of AGO2 was detected by M1-SUB pull-down/WB. **f** Gliotoxin reduced AGO2 M1-Ubi. 293T cells co-transfected with indicated plasmids were treated with Gliotoxin (5 μM) for indicated times. AGO2 M1-Ubi was determined by M1-SUB pull-down/WB. **g** UBL domain of HOIL-1L was essential for AGO2 M1-Ubi. HOIL-1L functional domain mutants (see Fig. 2**l**), HOIP and AGO2 were co-transfected into 293T cells. M1-Ubi of AGO2 was determined by M1-SUB pull-down/WB. **h** OTULIN decreased AGO2 M1-Ubi. 293T cells were co-transfected with indicated plasmids, and M1-Ubi of AGO2 was analyzed by IP/WB. **i**, **j** Knockdown of OTULIN increased AGO2 M1-Ubi. OTULIN and control siRNAs were co-transfected with Myc-AGO2 and Ub-7KR into 293T cells. M1-Ubi of AGO2 was detected by IP/WB with antibody 1E3 clone (**i**), or by M1-SUB pull-down/WB (**j**). **k** OTULIN but not mutants OTULIN (-W96A,-C129A), inhibited AGO2 M1-Ubi. OTULIN or indicated OTULIN mutants (W96A shields its interaction with M1-Ubi chain, and C129A blocks its enzymatic activity) were transfected into 293T cells. M1-Ubi of AGO2 was detected by and IP/WB. **l-n** Reshaped interaction of HOIP with OTULIN regulated AGO2 M1-Ubi. Myc-AGO2 and Ub-7KR were co-transfected with Flag-HOIP-WT or Flag-HOIP-mutants (Y82A, Y85/N94A and Y82A/N102D mutations of HOIP can decrease its association with OTULIN) and Flag-OTULIN-WT or Flag-OTULIN-mutation (Y56F mutation of OTULIN can enhance its interaction with HOIP) into 293T cells. M1-Ubi of AGO2 was detected by M1-SUB pull-down/WB (**l**, **n**), or by IP/WB with antibody LUB9 clone (**m**).

Supplementary Fig. 5

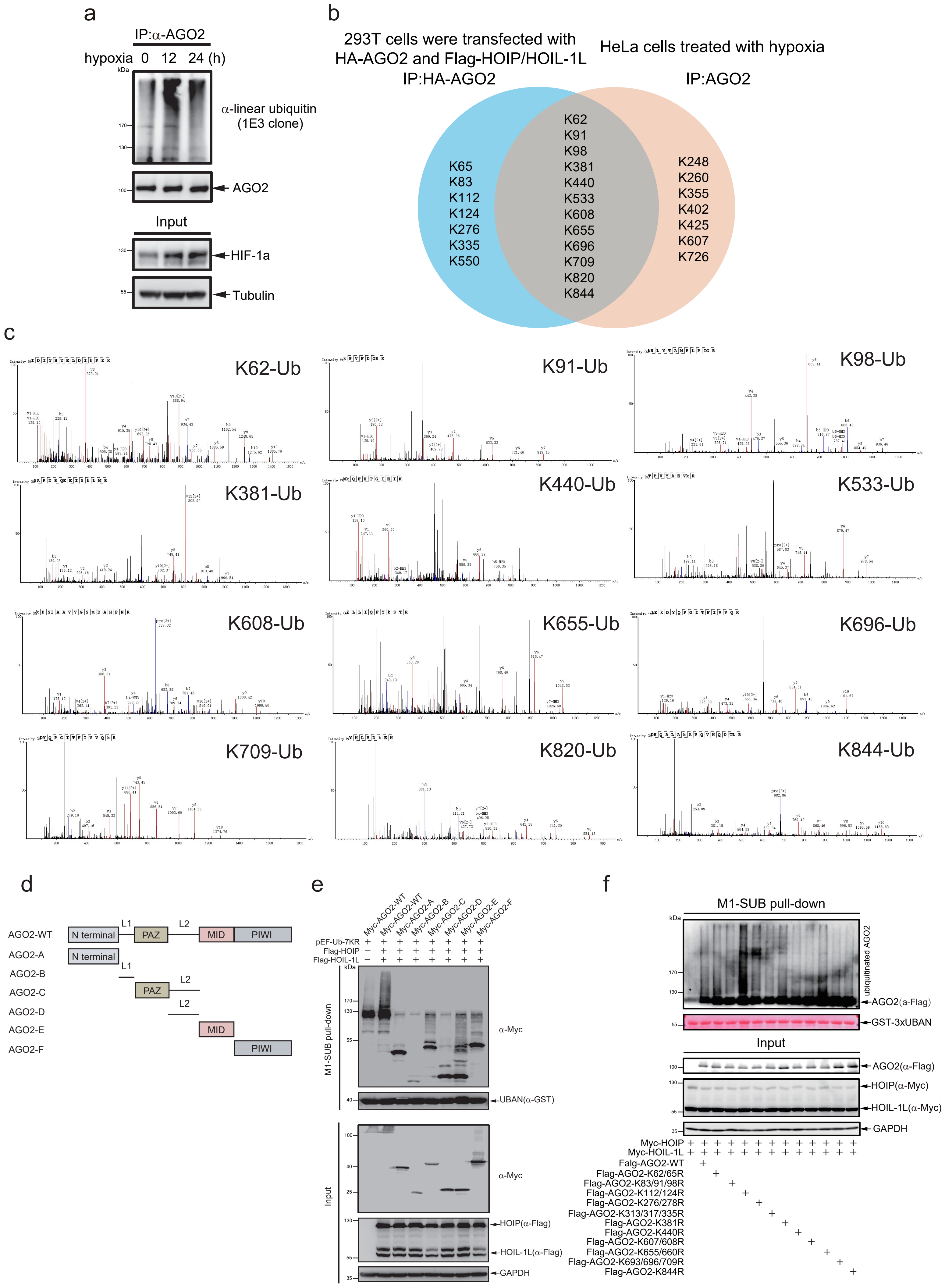

Supplementary Fig. 5 AGO2 is Met1-linear ubiquitinated at multiple sites. **a** Hypoxia promoted M1-Ubi of AGO2. HeLa cells were treated with hypoxia as indicated times and then lysed in the denaturing lysis buffer. Subsequently, cell lysates were diluted with lysis buffer for following immunoprecipitation with anti-AGO2 antibody, M1-Ubi of AGO2 was analyzed by Western blotting. **b** Overlapping of AGO2 ubiquitinated lysine residues in HeLa cells treated with hypoxia, and in 293T cells transfected with HA-AGO2, Flag-HOIP and Flag-HOIL-1L through IP-MS (mass spectrometry) analysis. HeLa cells treated with hypoxic conditions were performed for IP with as **a,** and 293T cells transfected with HA-AGO2, Flag-HOIP and Flag-HOIL-1L were conducted by IP with anti-HA antibody, respectively. Then the IPed samples were run on SDS-PAGE gel for following coomassie brilliant blue staining, gels containing M1-linear ubiquitinated AGO2 were cut and digested for MS analysis. **c** Overlapped multiple lysines for M1-Ubi of AGO2 were identified in 293T cells transfected with HA-AGO2, Flag-HOIP and Flag-HOIL-1L by mass spectrometry (MS) analysis. **d** The functional domain structures of human AGO2 and its truncated forms. **e** M1-Ubi sites of AGO2 were mainly located in PAZ, MID and PIWI domains. AGO2-WT or truncated AGO2 forms were individually co-transfected with HOIP and HOIL-1L into 293T cells. M1-Ubi of AGO2 and its truncated forms was determined by M1-SUB pull-down/WB. **f** Detection of potential M1-Ubi sites of AGO2. Flag-AGO2-WT and different mutants were co-transfected with Myc-HOIP and Myc-HOIL-1L into 293T cells, respectively. AGO2 M1-Ubi was detected by M1-SUB pull-down /WB.

Supplementary Fig. 6

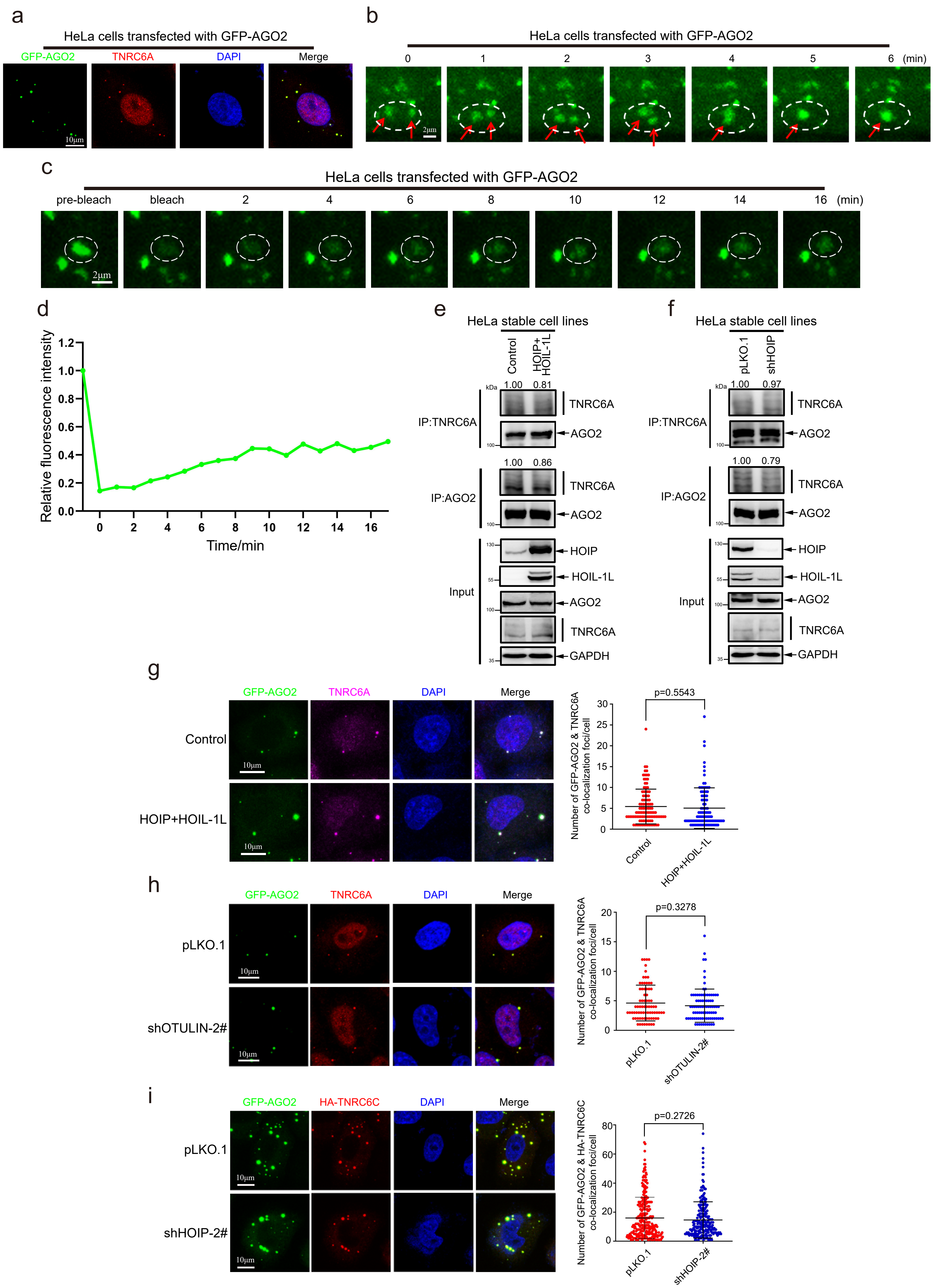

Supplementary Fig. 6 M1-Ubi of AGO2 does not affect the formation of miRISC. **a** Punctum formation of GFP-AGO2/TNRC6 in miRISC. GFP-AGO2 was transfected into HeLa cells, and the co-localization of GFP-AGO2 (green) with TNRC6A/GW182 (red) was observed by immunofluorescence staining. **b** Live cell images showing fusion process of two GFP-AGO2 foci in cytoplasm in HeLa cells transfected with GFP-AGO2 over time. **c**, **d** Representative live cell images of GFP-AGO2 foci in the FRAP experiments (**c**) and FRAP recovery curves (**d**). **e**, **f** Either LUBAC expression or HOIP knockdown did not affect the interaction of AGO2 with TNRC6A. HeLa cells with stably expressing HOIP/HOIL-1L (**e**) or HOIP knockdown by shRNA (**f**) were lysed for co-IP with anti-AGO2 or anti-TNRC6A antibody, respectively, and AGO2 association with TNRC6A was determined with WB. **g-i** HOIP/HOIL-1L expression, HOIP knockdown or OTULIN knockdown did not affect the formation of AGO2/TNRC6 in miRISC. HeLa cells with stably expressing HOIP and HOIL-1L (**g**), or knocking down OTULIN (**h**) or HOIP (**i**) by shRNAs were transfected with GFP-AGO2 and HA-TNRC6C. The co-localization of GFP-AGO2 with TNRC6A or with HA-TRNC6C was determined by immunofluorescence staining and the number of GFP-AGO2/TNRC6A or GFP-AGO2/HA-TNRC6C co-localization foci per cell were calculated. Data were mean ± s.d., and *P*-values were calculated by unpaired two-sided t-test.

Supplementary Fig. 7

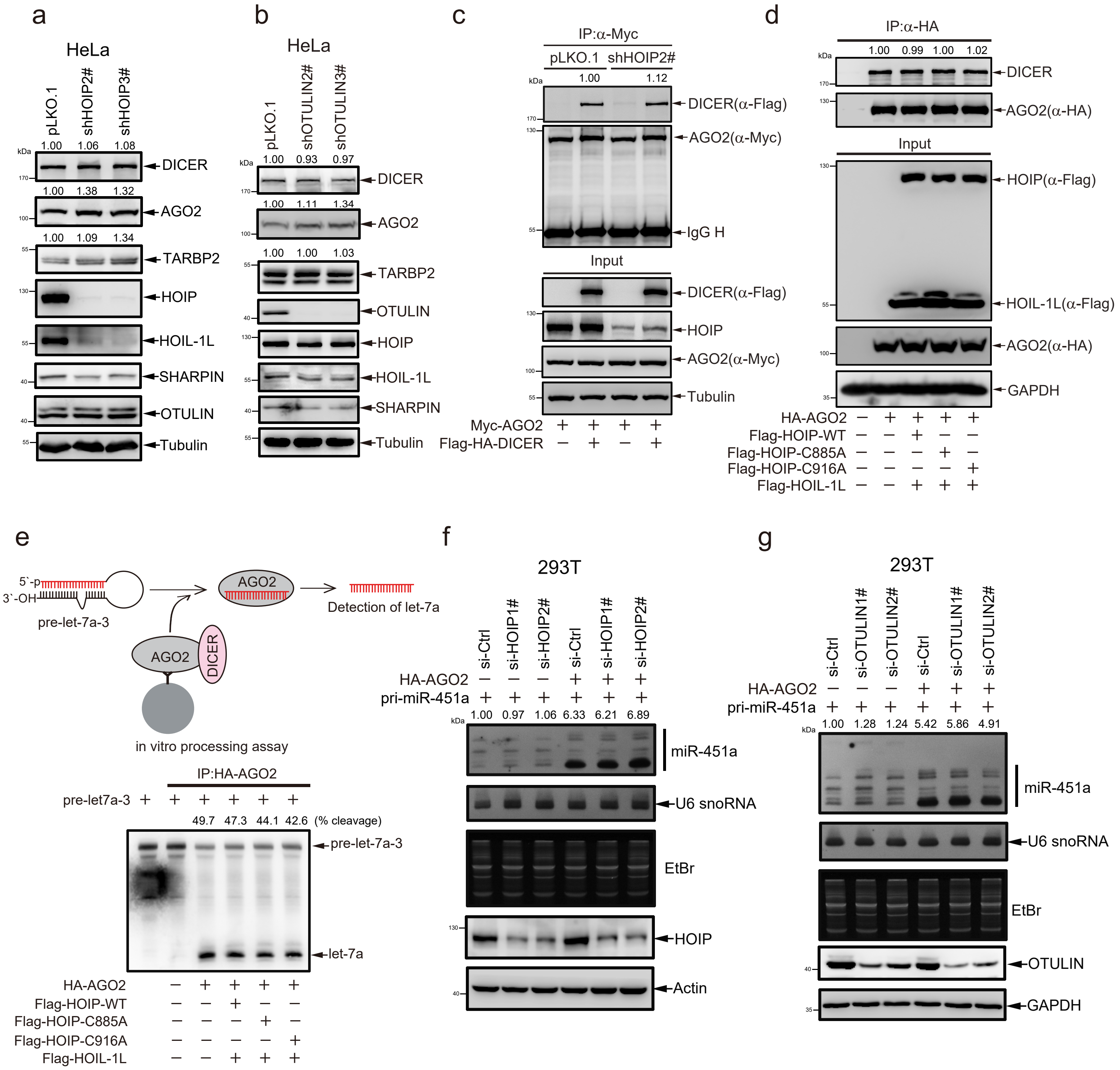

Supplementary Fig. 7 M1-Ubi of AGO2 has little effect on mature miRNA biogenesis. **a**, **b** Knockdown of either HOIP or OTULIN did not influence the expression levels of core proteins/enzymes in the miRNA pathway. HOIP (**a**) or OUTLIN (**b**) was stably knocked down by shRNA in HeLa cells, the expression levels of DICER, AGO2 and TARBP2 in the miRNA pathway were detected by WB. **c** Knockdown of HOIP had no effect on the association of AGO2 with DICER. Stable HeLa cells knocking down HOIP by shRNA were co-transfected with Myc-AGO2 and Flag-HA-DICER. Lysates were used for co-IP/WB detection. **d** M1-Ubi of AGO2 did not affect its interaction with DICER. HA-AGO2 and Flag-HOIL-1L were co-transfected with Flag-HOIP-WT, -C885A or -C916A into 293T cells. The interaction of endogenous of DICER with HA-AGO2 was determined by IP/WB. **e** M1-Ubi of AGO2 did not affect pre-let-7a-3 processing. HA-AGO2 and Flag-HOIL-1L were co-transfected with Flag-HOIP-WT, -C885A or -C916A into 293T cells. Lysates were used for IP with anti-HA antibody, and the beads enriching HA-AGO2 were incubated with purified biotin-tagged pre-let-7a-3 for *in vitro* processing assay. Processed mature let-7a and unprocessed pre-let-7a-3 were detected by Northern blotting analysis. **f**, **g** Knockdown of either HOIP or OUTLIN did not influence the biogenesis of mature miR-451a. 293T cells were co-transfected pri-miR-451a, HA-AGO2 with control vector, HOIP siRNA (**f**) or OTULIN siRNA (**g**). The expression levels of HOIP and OTULIN were examined by WB, and the biogenesis of miR-451a was determined by Northern blotting analysis.

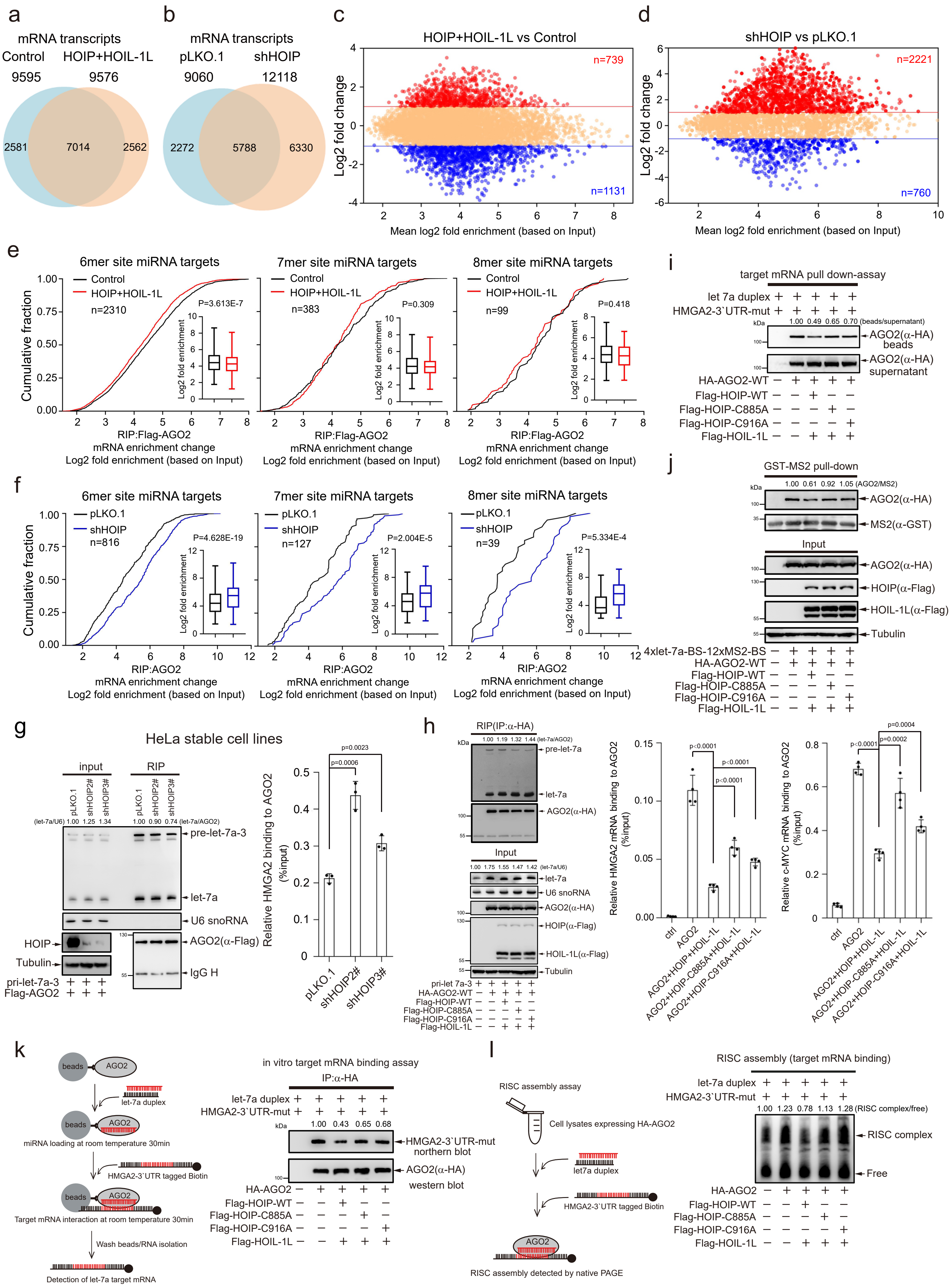

a
mRNA transcripts
Control HOIP+HOIL-1L
9595 9576
2581 7014 2562
b
mRNA transcripts
pLKO.1 shHOIP
9060 12118
2272 5788 6330
c
HOIP+HOIL-1L vs Control
n=739
n=1131
Log2 fold change
Mean log2 fold enrichment (based on Input)
d
shHOIP vs pLKO.1
n=2221
n=760
Log2 fold change
Mean log2 fold enrichment (based on Input)
e
6mer site miRNA targets
7mer site miRNA targets
8mer site miRNA targets
Control
HOIP+HOIL-1L
n=2310
n=383
n=99
P=3.613E-7
P=0.309
P=0.418
Cumulative fraction
Log2 fold enrichment
RIP:Flag-AGO2
mRNA enrichment change
Log2 fold enrichment (based on Input)
f
6mer site miRNA targets
7mer site miRNA targets
8mer site miRNA targets
pLKO.1
shHOIP
n=816
n=127
n=39
P=4.628E-19
P=2.004E-5
P=5.334E-4
RIP:AGO2
mRNA enrichment change
Log2 fold enrichment (based on Input)
g
HeLa stable cell lines
input RIP
pLKO.1 shHOIP2# shHOIP3#
(let-7a/U6) 1.00 1.25 1.34
1.00 0.90 0.74 (let-7a/AGO2)
pre-let-7a-3
let-7a
U6 snoRNA
HOIP
Tubulin
AGO2(α-Flag)
IgG H
pri-let-7a-3 + + +
Flag-AGO2 + + +
Relative HMGA2 binding to AGO2 (%input)
p=0.0023
p=0.0006
pLKO.1 shHOIP2# shHOIP3#
h
RIP(IP:α-HA)
1.00 1.19 1.32 1.44 (let-7a/AGO2)
pre-let-7a
let-7a
AGO2(α-HA)
Input
1.00 1.75 1.55 1.47 1.42 (let-7a/U6)
let-7a
U6 snoRNA
AGO2(α-HA)
HOIP(α-Flag)
HOIL-1L(α-Flag)
Tubulin
pri-let 7a-3
HA-AGO2-WT
Flag-HOIP-WT
Flag-HOIP-C885A
Flag-HOIP-C916A
Flag-HOIL-1L
Relative HMGA2 mRNA binding to AGO2 (%input)
Relative c-MYC mRNA binding to AGO2 (%input)
p<0.0001
p=0.0004
p=0.0002
ctrl
AGO2
AGO2+HOIP+HOIL-1L
AGO2+HOIP-C885A+HOIL-1L
AGO2+HOIP-C916A+HOIL-1L
i
target mRNA pull down-assay
let 7a duplex + + + + +
HMGA2-3`UTR-mut + + + + +
1.00 0.49 0.65 0.70 (beads/supernatant)
AGO2(α-HA) beads
AGO2(α-HA) supernatant
HA-AGO2-WT − + + + +
Flag-HOIP-WT − − + − −
Flag-HOIP-C885A − − − + −
Flag-HOIP-C916A − − − − +
Flag-HOIL-1L − − + + +
j
GST-MS2 pull-down
1.00 0.61 0.92 1.05 (AGO2/MS2)
AGO2(α-HA)
MS2(α-GST)
Input
AGO2(α-HA)
HOIP(α-Flag)
HOIL-1L(α-Flag)
Tubulin
4xlet-7a-BS-12xMS2-BS − + + + +
HA-AGO2-WT − + + + +
Flag-HOIP-WT − − + − −
Flag-HOIP-C885A − − − + −
Flag-HOIP-C916A − − − − +
Flag-HOIL-1L − − + + +
k
beads AGO2
let-7a duplex
miRNA loading at room temperature 30min
HMGA2-3`UTR tagged Biotin
Target mRNA interaction at room temperature 30min
Wash beads/RNA isolation
Detection of let-7a target mRNA
in vitro target mRNA binding assay
IP:α-HA
let-7a duplex + + + + +
HMGA2-3`UTR-mut + + + + +
1.00 0.43 0.65 0.68
HMGA2-3`UTR-mut northern blot
AGO2(α-HA) western blot
HA-AGO2 − + + + +
Flag-HOIP-WT − − + − −
Flag-HOIP-C885A − − − + −
Flag-HOIP-C916A − − − − +
Flag-HOIL-1L − − + + +
l
RISC assembly assay
Cell lysates expressing HA-AGO2
let7a duplex
HMGA2-3`UTR tagged Biotin
RISC assembly detected by native PAGE
RISC assembly (target mRNA binding)
let-7a duplex + + + + +
HMGA2-3`UTR-mut + + + + +
1.00 1.23 0.78 1.13 1.28 (RISC complex/free)
RISC complex
Free
HA-AGO2 − + + + +
Flag-HOIP-WT − − + − −
Flag-HOIP-C885A − − − + −
Flag-HOIP-C916A − − − − +
Flag-HOIL-1L − − + + +

Supplementary Fig. 8 AGO2 M1-Ubi impairs its recruiting miRNA-targeted mRNAs. **a-f** LUBAC reduced mRNA transcripts bound to AGO2. Stable HeLa-Flag-AGO2 cells expressing HOIP and HOIL-1L (**a**, **c**) and HeLa cells knocking down HOIP (**b**, **d**) were measured by RIP-Seq, respectively. Overlapping (**a**, **b**) and scatter plots (**c**, **d**) of mRNA transcripts bound to AGO2, respectively. Cumulative fraction analysis of targeted mRNA transcripts bound to AGO2, which were complementary paired with 6mer, 7mer and 8mer seed sequences of the top 10 non-difference miRNAs, through RIP-Seq in HeLa-Flag-AGO2 cells expressing LUBAC (**e**, n=2310 6mer mRNA transcripts, n=383 7mer mRNA transcripts, n=99 8mer mRNA transcripts) and HeLa cells knocking down HOIP (**f**, n=816 6mer mRNA transcripts, n=127 7mer mRNA transcripts, n=39 8mer mRNA transcripts). In box plots, the lines represent the median, first and third quartiles, the whiskers denote the minima and maxima; *P*-values for cumulative fraction analysis were calculated using a two-sided Mann-Whitney U test (**e**, **f**). **g**, **h** M1-Ubi of AGO2 did not affect its recruiting let-7a but inhibited its recruiting let-7-targeted mRNAs. HeLa cells knocking down of HOIP (**g**) and 293T cells (**h**) transfected with indicated plasmids were lysed for RIP assays. The RIP efficiency were determined by WB, and let-7a associated with AGO2 was detected by Northern blotting analysis. *HMGA2* and *c-MYC* mRNAs bound to AGO2 were measured by qRT-PCR. Data were mean ± s.d., n=3 (g) or n=4 (h) biologically independent samples, and *P*-values were determined by unpaired two-sided t-test. **i**, **j** M1-Ubi of AGO2 attenuated its associations with let-7a-targeted mRNA. 293T cells were transfected with indicated plasmids, and then cells were lysed for the target mRNA pull-down assay (**i**) and GST-MS2 pull-down assay (**j**). RNA bound fraction (beads) and unbound fraction (supernatant) were detected by WB (**i**). **k** LUBAC inhibited HMGA2-3`-UTR-mut interacting with AGO2. For *in vitro* target mRNA binding assay, 293T cells transfected with indicated plasmids were lysed for IP, followed by incubation with let-7a duplex and purified HMGA2-3`UTR-mut tagged by biotin. The HMGA2-3`-UTR-mut bound to AGO2 was examined by Northern blotting analysis. **l** LUBAC impaired HMGA2-3`-UTR-mut loading to the miRISC complex. 293T cells were transfected with indicated plasmids, followed by *in vitro* RISC assembly assay (procedure, left panel) with let-7a mimics and HMGA2-3`-UTR-mut. The miRISC complex was conducted by native gel and measured by Northern blotting.

Supplementary Table 1. Primer or oligonucleotide sequences were used in this study

The sequences of siRNA, shRNA and miRNA primers for qRT-PCR		
siRNA		
Negative control	sense	UUCUCCGAACGUGUCACGUTT
	anti-sense	ACGUGACACGUUCGGAGAATT
si-HOIP-1#	sense	CCUAGAACCUGAUCUUGCATT
	anti-sense	UGCAAGAUCAGGUUCUAGGTT
si-HOIP-2#	sense	GGCGUGGUGUCAAGUUUAATT
	anti-sense	UUAAACUUGACACCACGCCTT
si-OTULIN-1#	sense	GACUGAAAUUUGAUGGGAATT
	anti-sense	UUCCCAUCAAAUUUCAGUCTT
si-OTULIN-2#	sense	CAAAUGAGGCGGAGGAAUATT
	anti-sense	UAUUCCUCCGCCUCAUUUGTT
MiRNA mimics Negative control	sense	UUGUACUACACAAAAGUACUG
	anti-sense	GUACUUUUGUGUAGUACAAUU
let-7a mimics	sense	UGAGGUAGUAGGUUGUAUAGUU
	anti-sense	CUAUACAACCUACUACCUCAUU
shRNA		
HOIP shRNA1#	Forword	CCGGTTAATCCTGCAAGTGCTCATTTCTCGAGAAATGAGCACTTGCAGGATTATTTTTG
	Reverse	AATTCAAAAATAATCCTGCAAGTGCTCATTTCTCGAGAAATGAGCACTTGCAGGATTAA
HOIP shRNA2#	Forword	CCGGTGCGTGGTGTCAAGTTTAATAACCTCGAGGTTATTAAACTTGACACCACGCTTTTTG
	Reverse	AATTCAAAAAGCGTGGTGTCAAGTTTAATAACCTCGAGGTTATTAAACTTGACACCACGCA
HOIP shRNA3#	Forword	CCGGTGCACACTACAAAGAGTATCTTCTCGAGAAGATACTCTTTGTAGTGTGCTTTTTTG
	Reverse	AATTCAAAAAGCACACTACAAAGAGTATCTTCT

		CGAGAAGATACTCTTTGTAGTGTGCA
SHARPIN shRNA1#	Forword	CCGGTGGTGTTCTCAGAGCTCGGTTTCCTCGAG GAAACCGAGCTCTGAGAACAC CTTTTTG
	Reverse	AATTCAAAAAGGTGTTCTCAGAGCTCGGTTTCC TCGAGGAAACCGAGCTCTGAGAACACCA
SHARPIN shRNA2#	Forword	CCGGTGCTGTCCTTCCTGCACCTTCATCTCGAGA TGAAGGTGCAGGAAGGACAG CTTTTTG
	Reverse	AATTCAAAAAGCTGTCCTTCCTGCACCTTCATCT CGAGATGAAGGTGCAGGAAGGACAGCA
OTULIN shRNA1#	Forword	CCGGTGGCATCAGAACCGAGATTAAGCTCGAGC TTAATCTCGGTTCTGATGCCTTTTTG
	Reverse	AATTCAAAAAGGCATCAGAACCGAGATTAAGCT CGAGCTTAATCTCGGTTCTGATGCCA
OTULIN shRNA2#	Forword	CCGGTGAACAGGTTGAAATGTTCCTTCTCGAGA AGGAACATTTCAACCTGTTCTTTTTG
	Reverse	AATTCAAAAAGAACAGGTTGAAATGTTCCTTCT CGAGAAGGAACATTTCAACCTGTTCA
OTULIN shRNA3#	Forword	CCGGTCCCTTTAGTAGTAACGGGTTTCTCGAGA AACCCGTTACTACTAAAGGGTTTTTG
	Reverse	AATTCAAAAACCCTTTAGTAGTAACGGGTTTCTC GAGAAACCCGTTACTACTAAAGGGA
HOIP, HOIL-1L, OTULIN and AGO2 mutations sequences		
HOIP C885A	Forword	GCCATGCACTTTCACTGTACCCAG
	Reverse	GCCTCCTCGGGCCAGGGCGT
HOIP C916A	Forword	GCCAGGGTGAAAAAGTCCCTGCAC
	Reverse	GTTAGGCTCTGGACATTTAT
HOIP Y82A	Forword	GCCGGCCGCAACCTTCTCAGCCC
	Reverse	TTTCTCCAGGATGTTCAGAG
HOIP N102D	Forword	GACCCTGTCTTTCGCAGCACGGT
	Reverse	ATTAAACTTGACACCACGCC
HOIP Y84A/Y93A	Forword	CAGCGGCCTCGGGCCTGGCGTGGTGTCAAGTTT
	Reverse	AGGGCTGAGAAGGGCGCGGCCGTATTTCTCCAG
HOIL-1L C460A	Forword	GCCGACTGGATCCGCTGCACCGT
	Reverse	GCCGTCCTTCTTCTGTACCAC
OTULIN W96A	Forword	GCGAGAGGAAATACACAGAAAGC
	Reverse	TTCTTTTTTGCAGTAGTCCAT
OTULIN C129A	Forword	GCTGCACTGAGGGCCACGCT
	Reverse	GTAATTATCACCACGGAC

OTULIN Y56F	Forword	TTCCGTGCTGCAGATGAAATAG
	Reverse	CATGTCCTCCTCATGCTCGG
AGO2 K62/65R	Forword	GAGCGGTGCCCGAGGAGAGTTAAC
	Reverse	TGGCCGGATATCCAATTCATAATG
AGO2 K83/K91/K98R	Forword	CGGCCCGTGTTTGACGGCAGGCGGAATCTATAC
	Reverse	CCGATCCCCAAAGATCTGTGTCCGAAAGTGCTG
AGO2 K112/124R	Forword	GCTGCCAGGAGAAGGCCGGGATCGCA
	Reverse	GTGACCTCCAGCTCCACCCGGTCCCTC
AGO2 K212R	Forword	CGAATGATGCTGAATATTGATGT
	Reverse	CCAGAGAGAAGGCCGGACGG
AGO2 K226R	Forword	CGGGCACAGCCAGTAATCGAGTT
	Reverse	GTAAAACGCTGTTGCTGACA
AGO2 K276/278R	Forword	CGGAGGCGGTACCGCGTCTGCAATG
	Reverse	CATCTGCCCACAGTGCGTTATCTC
AGO2 K313/317/335 R	Forword	CCACCTCCCATGTTTACAAGTCGGACAGGAGCA GCGACACACCT
	Reverse	GGGTAGCGCAGAACCAACCGGTGCCTGTC CCGGAAATAC
AGO2 K381R	Forword	CGGTTGATGCGAAGTGCAAGTTTC
	Reverse	GCTAATCTCTTCTTGCCGATC
AGO2 K440R	Forword	AGACAGTTCCACACGGGCAT
	Reverse	GTTCCGCATGTCCCAGACGC
AGO2 K533R	Forword	CGGCGCGTGGGAGACACGGTGCT
	Reverse	GACCTCGGCGTACACGGGCGTCT
AGO2 K550R	Forword	CGGAACGTGCAGAGGACCACGC
	Reverse	CATCTGCACGCACTGCGTGGC
AGO2 K550/566R	Forword	GACCCTGTCCAACCTCTGCCTGCGGATCAACGT CAAGCTGGGAGGCG
	Reverse	TGTGGCGTGGTCCTCTGCACGTTCCGCATCTGC ACGCACTGCGT
AGO2 K607/608R	Forword	AGAAGACCCTCCATTGCCGCCGTG
	Reverse	CCCGGCGGGGGGTGAGTGA
AGO2 K655/660R	Forword	CGCTTCCGGCCCACCCGCATCATCTTCT
	Reverse	CGTGGACCGGTAGAACTGGATGAGGA
AGO2 K693/696/709 R	Forword	CGGGATCACCTTCATCGTGGTGCAGCGGAGGCA CCACACC
	Reverse	GGCTGGTAGTCCCGTTCTAGCCGGATACAGGCC

		TCACGGA
AGO2 K820R	Forword	AGAGAACATGACAGTGCTGA
	Reverse	ATCCACCAGGTGGTACCTGG
AGO2 K844R	Forword	GAGCGGTCCAGGTTCACCAAG
	Reverse	TGGCCAGTGCTTGGTGGT
QRT-PCR premers		
HOIP	Forword	TTTACGCCAAGAATAAATGTCC
	Reverse	CTCCTTCTGTATCACTC
HOIL-1L	Forword	CTTCATTGACAACACCTACTC
	Reverse	TGAACTCATTGACATCATCCT
SHARPIN	Forword	CACTGTTGCAGCTCTCCAGG
	Reverse	AAGTTCCCCGTCCATCTT
c-MYC	Forword	TCAAGAGGCGAACACACAAC
	Reverse	GGCCTT TTCATTGTTTTCCA
β-Actin	Forword	GCACAGAGCCTCGCCTT
	Reverse	GTTGTCGACGACGAGCG
NOMO1	Forword	TGCTCAGAGACGGCGAGAA
	Reverse	GCCCACTGCGGTGAAAGA
TBC1D20	Forword	TGAGATCAGACGAAAAGTGTGG
	Reverse	CATCTGCCGTAGGTTCTTCC
GFPT1	Forword	GGACAGCACAACCTGCCTTT
	Reverse	CAGCACTTGCATCAGAAGCAA
RAPH1	Forword	TCTTTGAGTATGGATGAGGCTG
	Reverse	GTGATGCTGGAATGGGAGG
PUM1	Forword	ACGAATGGCAGTGGAAGATAC
	Reverse	CGAGAGGAAGAGAAAAGAGTGC
GAPDH	Forword	CTCAAGGGCATCCTGGGCTA
	Reverse	ATGAGGTCCACCACCCTGTT
Northern blot probe sequences		
let-7a	sense	AACTATACAACCTACTACCTCA
miR-21	sense	TCAACATCAGTCTGATAAGCTA
miR-19b	sense	TCAGTTTTGCATGGATTTGCACA
U6	sense	TGTGCTGCCGAAGCGAGCAC
