# Supplementary material for: Hypoxia regulates overall mRNA homeostasis by inducing Met1-linked linear ubiquitination of AGO2 in cancer cells
Source: Nat Commun. 2021 Sep 13;12:5416. doi: 10.1038/s41467-021-25739-5 (PMC8438024; doi:10.1038/s41467-021-25739-5)
Supplement: Supplementary file 9 — Description of Additional Supplementary Files [file 41467_2021_25739_MOESM9_ESM.pdf]

Description of Additional Supplementary Files

Supplementary Data 1: Description

The mRNA transcripts bound to AGO2 in stable HeLa cells expressing Flag-AGO2 under hypoxia by RIP-seq.

Supplementary Data 2: Description

The miRNA expression profiles by miRNA-Seq in HeLa cells expressing Flag-AGO2 under hypoxia, stable HeLa-Flag-AGO2 cells expressing HOIP and HOIL-1L, and HeLa cells knocking down HOIP, respectively.

Supplementary Data 3: Description

RNA-Seq for mRNA expression profiles in HeLa cells expressing Flag-AGO2 under hypoxia and stable HeLa-Flag-AGO2 cells expressing HOIP and HOIL-1L, and the RNA-Seq for lncRNA expression profiles in stable HeLa-Flag-AGO2 cells expressing HOIP and HOIL-1L.

Supplementary Data 4: Description

Proteome associated by AGO2 under hypoxia in HeLa cells expressing Flag-AGO2 under hypoxia by MS.

Supplementary Data 5: Description

Identification of potential M1-Ubi sites of AGO2 by MS analysis. HeLa cells treated with hypoxia or 293T cells co-transfected with HA-AGO2 and Flag-HOIP/HOIL-1L were performed by IP/MS analysis.

Supplementary Data 6: Description

The mRNA transcripts bound to AGO2 in stable HeLaFlag-AGO2 cells expressing HOIP and HOIL-1L by RIP-seq.

Supplementary Data 7: Description

The mRNA transcripts bound to AGO2 in stable HeLa cells knocking down HOIP by RIP-seq.
