# Supplementary material for: Hypoxia regulates overall mRNA homeostasis by inducing Met1-linked linear ubiquitination of AGO2 in cancer cells
Source: Nat Commun. 2021 Sep 13;12:5416. doi: 10.1038/s41467-021-25739-5 (PMC8438024; doi:10.1038/s41467-021-25739-5)
Supplement: Supplementary file 10 — Reporting Summary [file 41467_2021_25739_MOESM10_ESM.pdf]

nature portfolio

Corresponding author(s): Jianxiu Yu

Last updated by author(s): Aug 14, 2021

Reporting Summary

Nature Portfolio wishes to improve the reproducibility of the work that we publish. This form provides structure for consistency and transparency in reporting. For further information on Nature Portfolio policies, see our Editorial Policies and the Editorial Policy Checklist.

Statistics

For all statistical analyses, confirm that the following items are present in the figure legend, table legend, main text, or Methods section.

n/a	Confirmed	
☐	☒	The exact sample size (*n*) for each experimental group/condition, given as a discrete number and unit of measurement
☐	☒	A statement on whether measurements were taken from distinct samples or whether the same sample was measured repeatedly
☐	☒	The statistical test(s) used AND whether they are one- or two-sided<br>*Only common tests should be described solely by name; describe more complex techniques in the Methods section.*
☒	☐	A description of all covariates tested
☒	☐	A description of any assumptions or corrections, such as tests of normality and adjustment for multiple comparisons
☒	☐	A full description of the statistical parameters including central tendency (e.g. means) or other basic estimates (e.g. regression coefficient) AND variation (e.g. standard deviation) or associated estimates of uncertainty (e.g. confidence intervals)
☐	☒	For null hypothesis testing, the test statistic (e.g. *F*, *t*, *r*) with confidence intervals, effect sizes, degrees of freedom and *P* value noted<br>*Give P values as exact values whenever suitable.*
☒	☐	For Bayesian analysis, information on the choice of priors and Markov chain Monte Carlo settings
☒	☐	For hierarchical and complex designs, identification of the appropriate level for tests and full reporting of outcomes
☐	☒	Estimates of effect sizes (e.g. Cohen's *d*, Pearson's *r*), indicating how they were calculated

Our web collection on statistics for biologists contains articles on many of the points above.

Software and code

Policy information about availability of computer code

Data collection	GraphPad Prism 8 (https://www.graphpad.com/scientific-software/prism/)<br>SPSS 25.0 (https://www.ibm.com/cn-zh/analytics/spss-statistics-software)<br>Fiji/ImageJ (https://imagej.net/Fiji)<br>Cutadapt (version 1.9.3, https://cutadapt.readthedocs.io/en/stable/#)<br>bowtie2 software (version 2.2.4, http://bowtie-bio.sourceforge.net/bowtie2/index.shtml)<br>hisat2 software (version 2.0.4, https://daehwankimlab.github.io/hisat2/)<br>MACS2 software (version 1.4.3)<br>Novoalign software (version 3.02.12)
Data analysis	GraphPad Prism 8 and SPSS 25.0 were performed for statistical calculation. Pearson's R value for IF staining images and western blot image quantitation were performed by Fiji/ImageJ. RIP-seq, RNA-seq and miRNA-seq were followed the software documentations, no special changes.

For manuscripts utilizing custom algorithms or software that are central to the research but not yet described in published literature, software must be made available to editors and reviewers. We strongly encourage code deposition in a community repository (e.g. GitHub). See the Nature Portfolio guidelines for submitting code & software for further information.

Data

Policy information about availability of data

All manuscripts must include a data availability statement. This statement should provide the following information, where applicable:

- Accession codes, unique identifiers, or web links for publicly available datasets
- A description of any restrictions on data availability
- For clinical datasets or third party data, please ensure that the statement adheres to our policy

High-throughput sequencing data of RIP-Seq, RNA-Seq and miRNA-Seq were provided as EXCEL profiles in Supplementary Data and were deposited at the Gene Expression Omnibus (GEO) repository with the accession number GSE158601. The mass spectrometry data were provided as EXCEL profiles in Supplementary Data and and uploaded to Integrated Proteome Resources (iProX) database with the accession number IPX0002506000. The remaining data supporting the findings of this paper are provided in the article. All data are also available from the corresponding author (J.Y. email:Jianxiu.Yu@gmail.com) upon reasonable request. Source analysis data are provided with this paper.

Field-specific reporting

Please select the one below that is the best fit for your research. If you are not sure, read the appropriate sections before making your selection.

☒ Life sciences ☐ Behavioural & social sciences ☐ Ecological, evolutionary & environmental sciences

For a reference copy of the document with all sections, see nature.com/documents/nr-reporting-summary-flat.pdf

Life sciences study design

All studies must disclose on these points even when the disclosure is negative.

Sample size	The experiments sample sizes in this study were chosen based on most studies with similar experimental procedures. At least three biological replicates of qRT-PCR and dual luciferase reporter assays were conducted and provided statistical significance.
Data exclusions	No data were excluded from analyses in the experiments.
Replication	All experiments of qRT-PCR, dual luciferase reporter assays, Western blot and northern blot were repeated at least three times and reliably reproduced.
Randomization	Molecular and cellular biology techniques were used in this study, which did not involve live organisms experiments, so there is no randomization techniques requirement.
Blinding	The results of molecular and cell biology experiments obtained is this article were by using objective quantitative methods, so the staffs were not blinded to sample allocation during expriments and had no idea of expecting outcome.

Reporting for specific materials, systems and methods

We require information from authors about some types of materials, experimental systems and methods used in many studies. Here, indicate whether each material, system or method listed is relevant to your study. If you are not sure if a list item applies to your research, read the appropriate section before selecting a response.

Materials & experimental systems

n/a	Involved in the study	
☐	☒	Antibodies
☐	☒	Eukaryotic cell lines
☒	☐	Palaeontology and archaeology
☒	☐	Animals and other organisms
☒	☐	Human research participants
☒	☐	Clinical data
☒	☐	Dual use research of concern

Methods

n/a	Involved in the study	
☒	☐	ChIP-seq
☒	☐	Flow cytometry
☒	☐	MRI-based neuroimaging

Antibodies

Antibodies used	Anti-AGO2, CST, Cat# 2897 (1:1000 dilution)<br>Anti-Myc, CST, Cat# 2276 (1:1000 dilution)<br>Anti-DICER, CST, Cat# 3363 (1:1000 dilution)<br>Anti-PTEN, CST, Cat# 9188 (1:1000 dilution)<br>Anti-GFP, CST, Cat# 2956 (1:200 dilution)

Anti-linear ubiquitin LUB9 clone, LifeSensors, Cat# AB130 (1:1000 dilution)
Anti-AGO2 11A9 clone, Millipore, Cat# MABE253 (1:1000 dilution)
Anti-linear ubiquitin 1E3 clone, Millipore, Cat# MABS199 (1:1000 dilution)
Anti-AGO2, Abcam, Cat# ab57113 (1:1000 dilution)
Anti-HOIL-1L, Abcam, Cat# ab38540 (1:1000 dilution)
Anti-TNRC6A, Abcam, Cat# ab84403 (1:50-1:100 dilution for immunofluorescence staining)
Anti-OTULIN, Abcam, Cat# ab151117 (1:1000 dilution)
Anti-DGCR8, Proteintech, Cat# 60084-1-Ig (1:1000 dilution)
Anti-TARBP2, Proteintech, Cat#15753-1-AP (1:1000 dilution)
Anti-His, Proteintech, Cat# 66005-1-Ig (1:1000 dilution)
Anti-GAPDH, Proteintech, Cat# 60004-1-Ig (1:5000 dilution)
Anti-Tubulin, Proteintech, Cat# 66031-1-Ig (1:5000 dilution)
Anti-SHARPIN, Proteintech, Cat# 14626-1-AP (1:1000 dilution)
Anti-HIF-1α, NOVUSBIO, Cat# NB-100-479 (1:1000 dilution)
Anti-Flag, Sigma, Cat# F1804 (1:1000 dilution)
Anti-HA, Covance, Cat# A448-101L (1:1000 dilution)
Normal mouse IgG, Santa Cruz Biotechnology, Cat# sc-2025
Anti-TNRC6A, Santa Cruz Biotechnology, Cat# sc-56314 (1:500 dilution, 1:50-1:100 dilution for immunofluorescence staining)
Anti-HOIL-1L, Santa Cruz Biotechnology, Cat# sc-393754 (1:1000 dilution, 1:50-1:100 dilution for immunofluorescence staining)
Anti-GST, CwbioTech, Cat# CW0084 (1:3000 dilution)
Anti-HOIP, sigma-aldrich Cat# SAB2102031 (1:1000 dilution)

Validation

All antibodies used in this study are commercially available and have been validated by manufacturer.Antibody validations and validation criteria are available on the following websites:
Anti-AGO2 https://www.cellsignal.cn/products/primary-antibodies/argonaute-2-c34c6-rabbit-mab/2897?site-search-type=Products&N=4294956287&Ntt=2897&fromPage=plp&_requestid=3053072
Anti-Myc https://www.cellsignal.cn/products/primary-antibodies/myc-tag-9b11-mouse-mab/2276?site-search-type=Products&N=4294956287&Ntt=2276&fromPage=plp
Anti-DICER https://www.cellsignal.cn/products/primary-antibodies/dicer-antibody/3363?site-search-type=Products&N=4294956287&Ntt=3363&fromPage=plp&_requestid=3078521
Anti-PTENhttps://www.cellsignal.cn/products/primary-antibodies/pten-d4-3-xp-rabbit-mab/9188?site-search-type=Products&N=4294956287&Ntt=9188&fromPage=plp&_requestid=3081892
Anti-GFP https://www.cellsignal.cn/products/primary-antibodies/gfp-d5-1-rabbit-mab/2956?site-search-type=Products&N=4294956287&Ntt=2956&fromPage=plp&_requestid=3081998
Anti-linear ubiquitin LUB9 clone https://lifesensors.com/product/ab130-linear-polyubiquitin-antibody-mab-clone-lub9/
Anti-AGO2 11A9 clone https://www.merckmillipore.com/CN/zh/product/Anti-Ago2-Antibody-clone-11A9,MM_NF-MABE253?bd=1#anchor_COA
Anti-linear ubiquitin 1E3 clone https://www.sigmaaldrich.cn/CN/zh/product/mm/mabs199?context=product
Anti-AGO2 https://www.abcam.cn/argonaute-2-antibody-2e12-1c9-bsa-and-azide-free-ab57113.html
Anti-HOIL-1L file:///C:/Users/hailong%20Zhang/Downloads/datasheet_38540%20(1).pdf
Anti-TNRC6A file:///C:/Users/hailong%20Zhang/Downloads/datasheet_84403%20(1).pdf
Anti-OTULIN https://www.abcam.cn/otulin-antibody-ab151117.html
Anti-DGCR8 http://www.ptgcn.com/products/DGCR8-Antibody-60084-1-Ig.htm
Anti-TARBP2 http://www.ptgcn.com/products/TRBP-Antibody-15753-1-AP.htm
Anti-His http://www.ptgcn.com/products/His-Tag-Antibody-66005-1-Ig.htm
Anti-GAPDH http://www.ptgcn.com/products/GAPDH-Antibody-60004-1-Ig.htm
Anti-Tubulin http://www.ptgcn.com/products/tubulin-Alpha-Antibody-66031-1-Ig.htm
Anti-SHARPIN http://www.ptgcn.com/products/SHARPIN-Antibody-14626-1-AP.htm
Anti-HIF-1α https://www.novusbio.com/products/hif-1-alpha-antibody-pack_nb100-905#supportresearch
Anti-Flag https://www.sigmaaldrich.cn/CN/zh/product/sigma/f1804?context=product
Anti-HA https://www.biolegend.com/en-us/products/purified-anti-ha-11-epitope-tag-antibody-11374
Normal mouse IgG https://www.scbt.com/p/normal-mouse-igg?requestFrom=search
Anti-TNRC6A https://www.scbt.com/p/gw182-antibody-4b6?requestFrom=search
Anti-HOIL-1L https://www.scbt.com/p/rbck1-antibody-h-1?requestFrom=search
Anti-GST https://www.cwbio.com/goods/index/id/10104
Anti-HOIP https://www.sigmaaldrich.cn/CN/zh/product/sigma/sab2102031?context=product

Eukaryotic cell lines

Policy information about cell lines

Cell line source(s)

HeLa, Stem Cell Bank, Chinese Academy of Sciences, Cat# TCHu187
H1299, Stem Cell Bank, Chinese Academy of Sciences, Cat# TCHu160
DU145, Stem Cell Bank, Chinese Academy of Sciences, Cat# TCHu222
HEK 293T, Stem Cell Bank, Chinese Academy of Sciences, Cat# GNHu17
A549, Stem Cell Bank, Chinese Academy of Sciences, Cat# TCHu150
PC3, Stem Cell Bank, Chinese Academy of Sciences, Cat# TCHu158
BPH1 cell was gotten from Prof. Simon W. Hayward of Vanderbilt University with MTA

Authentication

HeLa, H1299, DU145, HEK 293T, A549 and PC3 were purchased from Stem Cell Bank (Chinese Academy of Sciences. Shanghai. China) of which cell lines have been thoroughly tested and authenticated. Each cell line used in this study were observed by microscope to check the shape, growth condition and any contaminants, cells growth and proliferation were in well condition.

Mycoplasma contamination	The cell lines used in this study were not tested for mycoplasma contamination. However, all the cell lines we used were treatment with mycoplasma removal agent (MP, cat# 093050044) to eliminate mycoplasma in this study.
Commonly misidentified lines (See ICLAC register)	None
